# The P2X7R/NLRP3 inflammasome axis suppresses enthesis regeneration through inflammatory and metabolic macrophage-stem cell cross-talk

**DOI:** 10.1126/sciadv.adr4894

**Published:** 2025-04-25

**Authors:** Haihan Gao, Liren Wang, Yangbao Lyu, Haocheng Jin, Zhiqi Lin, Yuhao Kang, Ziyun Li, Xueying Zhang, Yuhan Jiang, Guoyang Zhang, Zaijin Tao, Xiaofeng Zhang, Bin Yang, Xingyu Bai, Xin Ma, Shen Liu, Jia Jiang

**Affiliations:** ^1^ Department of Orthopedic Surgery, Shanghai Sixth People’s Hospital Affiliated to Shanghai Jiao Tong University School of Medicine, Shanghai 200233, China.; ^2^ Regenerative Sports Medicine and Translational Youth Science and Technology Innovation Workroom, Shanghai Jiao Tong University School of Medicine, Shanghai 200020, China.; ^3^ Department of Orthopedic Surgery, Jinshan Branch of Shanghai Sixth People’s Hospital Affiliated to Shanghai Jiao Tong University School of Medicine, Shanghai 201500, China.; ^4^ National Key Laboratory of Advanced Micro and Nano Manufacture Technology, Shanghai Jiao Tong University, Shanghai 200240, China.

## Abstract

The regeneration of the enthesis remains a formidable challenge in regenerative medicine. However, key regulators underlying unsatisfactory regeneration remain poorly understood. This study reveals that the purinergic receptor P2X7 (P2X7R)/Nod-like receptor family protein 3 (NLRP3) inflammasome axis suppresses enthesis regeneration by amplifying IL-1β–mediated inflammatory cross-talk and suppressing docosatrienoic acid (DTA) metabolic cross-talk. NLRP3 inflammasomes were activated in macrophages following enthesis injury, thereby impairing the histological and functional recovery of the injured enthesis. Single-cell RNA sequencing (scRNA-seq) indicated that *Nlrp3* knockout attenuated pathological inflammation and ameliorated the detrimental effects of IL-1β signaling cross-talk. Furthermore, NLRP3 inflammasomes suppressed the secretion of anti-inflammatory cytokines (IL-10 and IL-13) and DTA. The NLRP3 inflammasome–mediated secretome reduced differentiation and migration of stem cells. Neutralizing IL-1β or replenishing docosatrienoic acid accelerated enthesis regeneration. Moreover, conditional knockout of *P2rx7* in myeloid cells attenuated NLRP3 inflammasome activation and facilitated enthesis regeneration. This study demonstrates that the P2X7R/NLRP3 inflammasome axis represents a promising therapeutic target for enthesis repair.

## Teaser

The P2X7R/NLRP3 inflammasome axis suppresses tendon-to-bone interface regeneration through inflammatory and metabolic cross-talk.

## INTRODUCTION

Heterogeneous interface tissues, such as the osteochondral and tendon-to-bone interfaces, are pivotal in bridging the transition between soft and hard tissues (*1*, *2*). Their primary function is to facilitate the transfer of stress from soft tissues to bone, thereby mitigating stress concentration and ensuring smooth joint movement (*1*, *2*). The tendon-to-bone interface, referred to as the enthesis, is a complex structure consisting of the tendon, uncalcified fibrocartilage, calcified fibrocartilage, and bone (*3*). The sophisticated structure of the enthesis underpins its function (*4*). Injuries to the enthesis, including rotator cuff tears and anterior cruciate ligament injuries, are prevalent among the elderly and physically active individuals (*5*). Unfortunately, because of the limited regenerative capacity of the enthesis, the intricate structure and function cannot be fully restored postinjury, resulting in an increased risk of reinjury (*6*, *7*). Therefore, identifying key factors that impede enthesis regeneration is critical in the field of regenerative medicine and essential for developing effective therapeutic strategies for enthesis injuries.

Tissue regeneration is manipulated by the local niche, comprising cellular components, inflammation, secreted factors, metabolites, and extracellular matrix, among others (*8*, *9*). Ideal regeneration depends on the harmonious orchestration of components in the niche (*10*, *11*). Inflammation, a crucial component of the niche, often acts as a double-edged sword in regeneration (*12*, *13*). Reparative inflammation definitively takes charge of regeneration, whereas pathological inflammation inevitably inhibits regeneration and leads to degeneration (*14*, *15*). Growing evidence indicates that inadequate enthesis regeneration is strongly associated with the pathological proinflammatory niche, characterized by an overabundance of proinflammatory macrophages (PIM) and cytokines, as well as stem cells with diminished regenerative abilities (*16*–*18*). Nevertheless, the specific mechanisms governing the cross-talk between macrophages and stem cells within this pathological niche, as well as its impact on enthesis regeneration, remain poorly understood.

Nod-like receptor family protein 3 (NLRP3) inflammasome, which is typically activated in immune cells such as macrophages following tissue injury, serves as a crucial bridge between inflammation and tissue regeneration (*19*, *20*). Upon activation, the NLRP3 inflammasome triggers the release of proinflammatory cytokines, including interleukin-1β (IL-1β) and IL-18, through gasdermin D (GSDMD)–mediated pyroptosis (*20*). IL-1β not only impairs the proliferation, migration, and differentiation of stem cells through inhibiting the Akt/GSK-3β/β-catenin pathway but also blunts the effectiveness of growth factors on the differentiation of stem cells and tissue regeneration (*21*, *22*). Beyond its role in cytokine production, the NLRP3 inflammasome has been implicated in reprogramming cellular metabolism and modulating the release of metabolites that influence regeneration (*23*). The activation of NLRP3 inflammasome initiates de novo prostaglandin E2 (PGE2) synthesis that accelerates the migration of fibroblasts and wound healing (*23*). Nonetheless, how the deficiency of NLRP3 inflammasomes modulates cellular metabolic processes is enigmatic. Previous studies have demonstrated that the nuclear factor κB (NF-κB) pathway, a key priming signal for NLRP3 inflammasome activation, is up-regulated in clinical samples of enthesis injury, and elevated expression of NLRP3 and caspase-1 has been observed in rodent models of enthesis injury (*24*, *25*). Despite these findings, the precise role of the NLRP3 inflammasome in orchestrating inflammation and mediating cross-talk between macrophages and stem cells during enthesis repair and regeneration is still unclarified.

Here, it was observed that activation of NLRP3 inflammasomes in macrophages suppressed both histological and functional recovery of the injured enthesis. Single-cell RNA sequencing (scRNA-seq) suggested that NLRP3 inflammasomes exacerbated inflammation and contributed to adverse IL-1β signaling cross-talk between macrophages and stem cells. Moreover, NLRP3 inflammasomes suppressed the secretion of anti-inflammatory cytokines, including IL-10 and IL-13, as well as unsaturated fatty acids such as DTA, which reduced the differentiation and migration of stem cells. Neutralizing IL-1β or replenishing DTA facilitated enthesis regeneration. In addition, conditional knockout (KO) of *P2rx7* in myeloid cells reduced the activity of NLRP3 inflammasomes and facilitated enthesis regeneration. This study elucidates that the P2X7R/NLRP3 inflammasome axis inhibits enthesis regeneration through exacerbating IL-1β inflammatory cross-talk and inhibiting proregenerative metabolic cross-talk between macrophages and stem cells, which may offer promising therapeutic avenues for the treatment of heterogeneous interface tissue injuries.

## RESULTS

### NLRP3 inflammasomes are activated in macrophages following enthesis injury

**Fig. 1.**
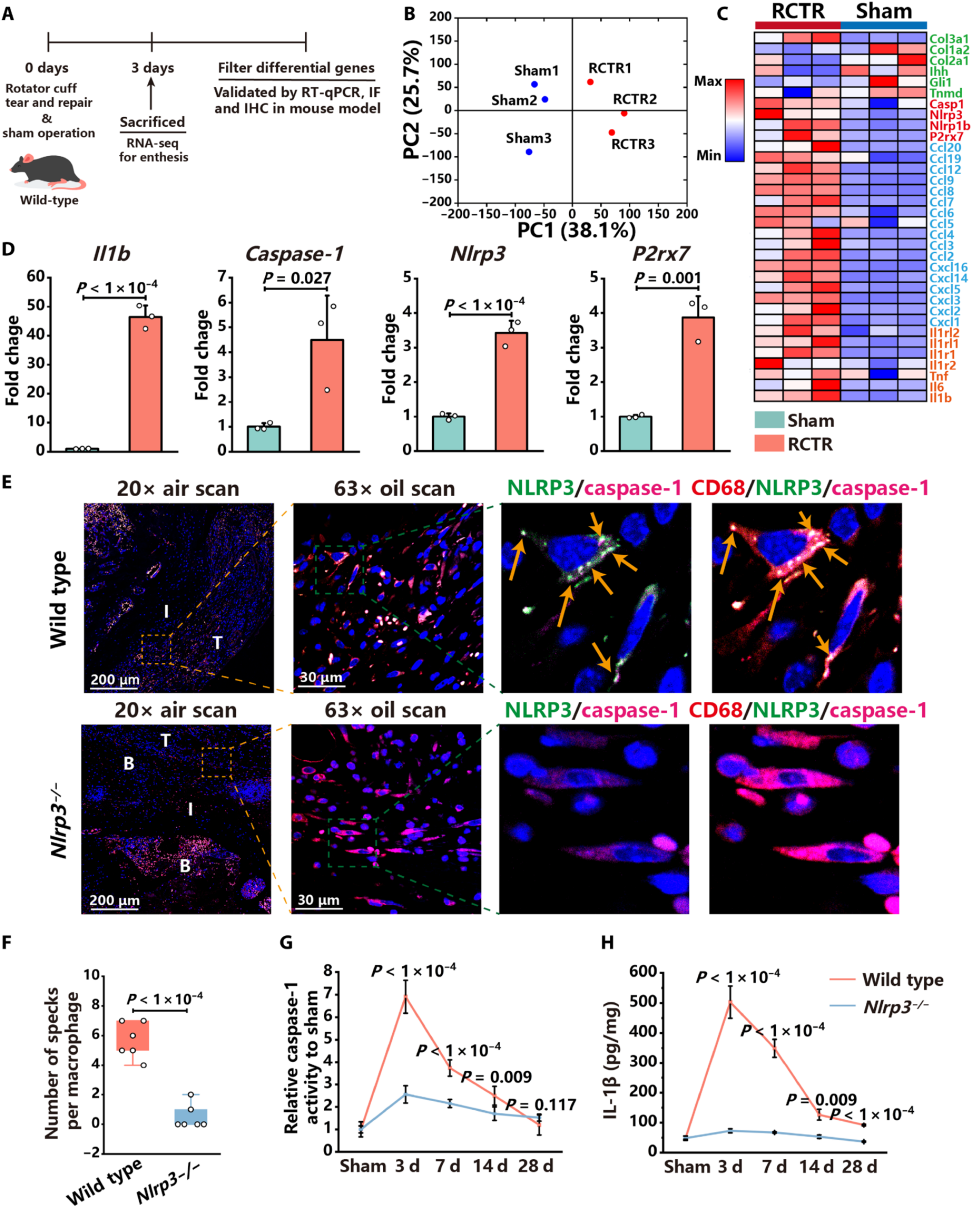
NLRP3 inflammasomes are activated in macrophages at the injured enthesis. (**A**) Schematic diagram of RNA-seq and validation of DEGs. (**B**) PCA of the sham operation and RCTR groups. (**C**) Heat map of DEGs between the sham operation and RCTR groups. (**D**) Relative mRNA expression levels of *Il1b*, *Caspase-1*, *Nlrp3*, and *P2rx7* in the enthesis of RCTR groups in comparison to sham operation groups. (**E**) Immunofluorescence (IF) staining of CD68 (red), NLRP3 (green), and caspase-1 (magenta) in the injured enthesis of wild-type and *Nlrp3^−/−^* mice at 3 dpi. Orange and green dashed squares represent enlarged images of the enthesis. Arrows indicate specks of NLRP3 and caspase-1. (**F**) Quantification of specks per macrophage in the injured enthesis of wild-type and *Nlrp3^−/−^* mice at 3 dpi. (**G** and **H**) The relative caspase-1 activity and IL-1β concentration of the enthesis in wild-type and *Nlrp3^−/−^* mice at 3, 7, 14, and 28 dpi. T, tendon; I, tendon-to-bone interface; B, bone. Data are presented as means ± SD. Statistical significance was determined using one-way analysis of variance (ANOVA) with Tukey’s multiple comparisons test. d, days.

To explore changes of enthesis gene profiles following injury, a murine enthesis injury model was established. RNA-seq of the injured enthesis was performed at 3 days postinjury (dpi), which is considered the inflammatory phase of enthesis regeneration (Fig. 1A) (*26*). Principal components analysis (PCA) revealed a notably distinct gene expression profile between the sham operation and rotator cuff tear and repair (RCTR) groups (Fig. 1B). The volcano plot showed 869 up-regulated and 740 down-regulated genes (fig. S1A). Gene Ontology (GO) analysis revealed that differentially expressed genes (DEGs) were enriched in macrophage migration, macrophage activation, response to IL-1, and regulation of IL-1β production (fig. S1B). Expression of genes related to inflammatory cytokines and receptors (*Il1b*, *Il6*, *Tnf*, *Il1r1*, etc.), chemokines (*Cxcl1*, *Cxcl2*, *Ccl2*, *Ccl3*, etc.), and NLRP3 inflammasomes (*Nlrp3*, *Casp1*, and *P2rx7*) was up-regulated, whereas those related to enthesis regeneration (*Tnmd*, *Gli1*, *Ihh*, *Col2a1*, and *Col1a2*) were down-regulated (Fig. 1C). The gene expression changes of *Nlrp3*, *Caspase-1*, *Il1b*, and *P2rx7* in the enthesis were validated by real-time quantitative polymerase chain reaction (RT-qPCR) and were consistent with RNA-seq (Fig. 1D). After the sham operation, neither IL-1β expression nor macrophage infiltration was observed in native enthesis (fig. S1, C and D). Moreover, the immunohistochemical (IHC) staining of IL-1β and its integrated optical density (IOD) revealed that the expression of IL-1β peaked at 3 dpi and gradually decreased to the base level at 28 dpi (fig. S1, C and E). Immunofluorescence staining of inducible nitric oxide synthase (iNOS)^+^ and CD68^+^ macrophages revealed that the number of PIM significantly increased at 3 dpi and gradually returned to baseline at 28 dpi (fig. S1, D and F). Above results reveal that enthesis injury leads to PIM infiltration and elevated expression of *Nlrp3*, *Caspase-1*, and *Il1b* in the mouse model.

We further investigated the expression of NLRP3 in various cell types. Immunofluorescence staining revealed that NLRP3 was primarily expressed in CD68^+^ macrophages that infiltrated the enthesis following injury (fig. S2A). The number of CD68^+^ and NLRP3^+^ macrophages peaked at 3 dpi in wild-type controls and gradually decreased to baseline levels at 28 dpi (fig. S2B). CD31^+^ endothelial cells, α-smooth muscle actin (α-SMA)^+^ myofibroblasts, tenomodulin (Tnmd)^+^ tenocytes, and Platelet-derived growth factor receptor alpha (Pdgfrα)^+^ stem cells exhibited negligible expression of NLRP3 (fig. S3, A to E). Subsequently, immunofluorescence staining, the relative caspase-1 activity, and the IL-1β concentration of the enthesis were used to evaluate the assembly and activation of NLRP3 inflammasomes. Immunofluorescence staining showed that colocalization of NLRP3 and caspase-1 formed specks, suggesting the assembly and activation of NLRP3 inflammasomes at 3 dpi (Fig. 1E). In contrast, almost no NLRP3 expression or caspase-1 specks were observed in the injured enthesis of *Nlrp3^−/−^* mice (Fig. 1, E and F). The relative caspase-1 activity significantly increased at 3 dpi and then progressively declined in both wild-type controls and *Nlrp3^−/−^* mice (Fig. 1G). However, the relative caspase-1 activity of wild-type controls was significantly higher than that of *Nlrp3^−/−^* mice at 3, 7, and 14 dpi (Fig. 1G). No notable disparity was observed in the relative caspase-1 activity between wild-type controls and *Nlrp3^−/−^* mice at 28 dpi (Fig. 1G). Enzyme-linked immunosorbent assay (ELISA) exhibited that the IL-1β concentration in the enthesis of wild-type controls significantly increased at 3 dpi and then gradually decreased back to baseline levels at 28 dpi (Fig. 1H). Compared to wild-type controls, the concentration of IL-1β in the enthesis was lower in *Nlrp3^−/−^* mice at all time points (Fig. 1H). Together, the above results indicate that NLRP3 inflammasomes are activated in infiltrated macrophages following enthesis injury.

### The activation of NLRP3 inflammasomes suppresses enthesis regeneration

The role of NLRP3 inflammasomes in enthesis regeneration was further investigated. First, the native enthesis in both wild-type controls and *Nlrp3^−/−^* mice was assessed to ensure that the absence of NLRP3 did not affect enthesis development. Hematoxylin and eosin (H&E ) and toluidine blue staining revealed that fibrocartilage cells at the enthesis in both wild-type controls and *Nlrp3^−/−^* mice were arranged in columns aligned with the direction of the tendon (fig. S4, A and B). In addition, a distinct tidemark was observed between the uncalcified and calcified fibrocartilage layers (fig. S4, A and B). Moreover, no differences were observed in bone volume/total volume (BV/TV), bone mineral density (BMD), or trabecular number (Tb. N) levels of the enthesis between wild-type controls and *Nlrp3^−/−^* mice (fig. S4, C to F). These results indicate that the absence of NLRP3 does not influence the native structure of the enthesis.

**Fig. 2.**
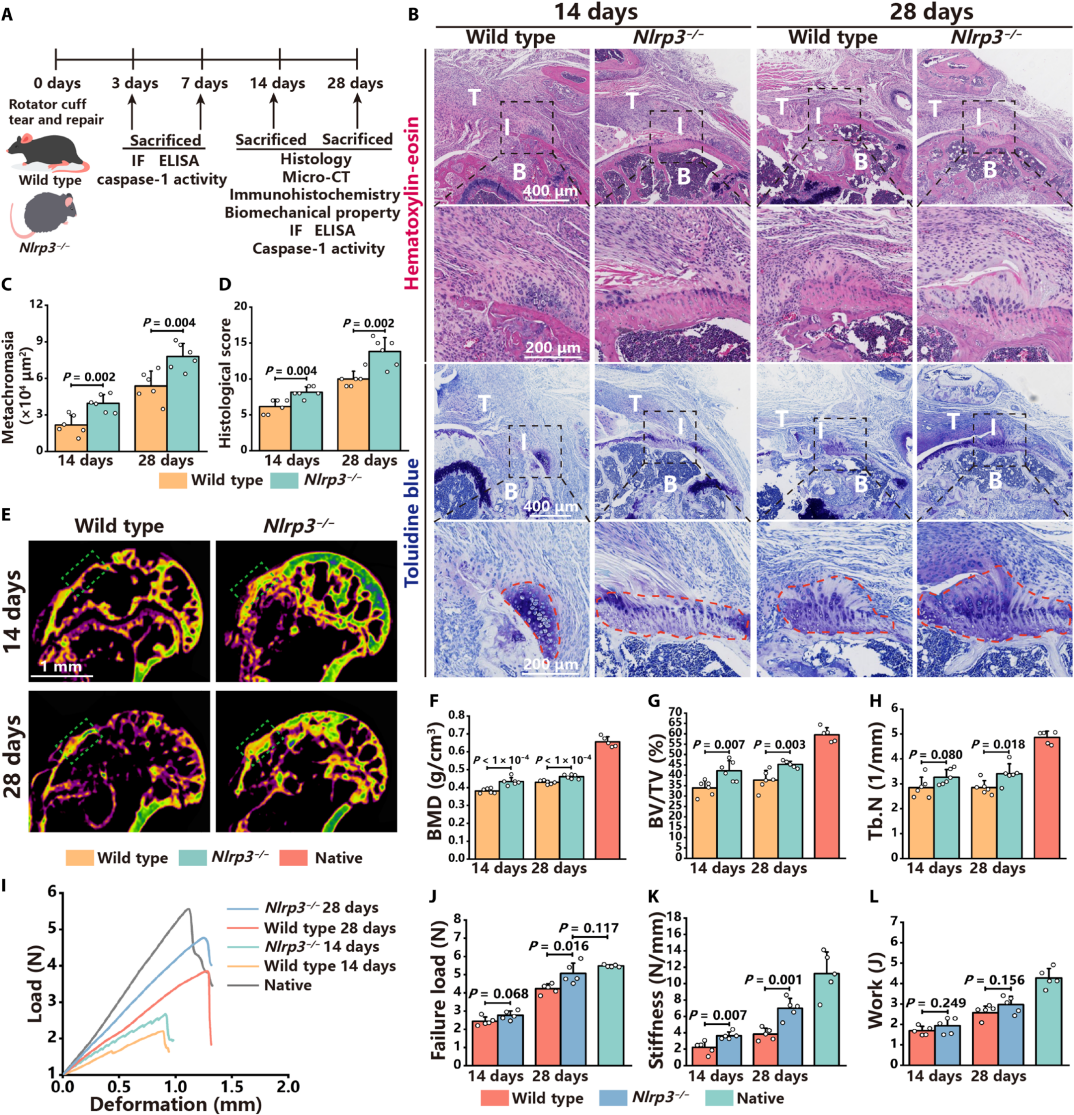
The activation of NLRP3 inflammasomes inhibits enthesis regeneration. (**A**) Schematic of animal experiments, in which the enthesis injury and the repair model were established in wild-type and *Nlrp3^−/−^* mice, with analyses at 3, 7, 14, and 28 dpi. (**B**) H&E and toluidine blue staining of the enthesis in wild-type and *Nlrp3^−/−^* mice at 14 and 28 dpi. Black dashed squares represent enlarged images of the enthesis. (**C** and **D**) Metachromasia area size and histological scores of the enthesis in wild-type and *Nlrp3^−/−^* mice at 14 and 28 dpi. (**E**) Micro-CT coronal views of the humerus of wild-type and *Nlrp3^−/−^* mice at 14 and 28 dpi. Green dashed squares represent the area of the enthesis. (**F** to **H**) Quantitative analysis of BMD, BV/TV, and Tb.N of the enthesis. (**I**) Deformation and load curves of the enthesis in wild-type and *Nlrp3^−/−^* mice at 14 and 28 dpi. (**J** to **L**) Failure load, stiffness, and work of the enthesis in wild-type and *Nlrp3^−/−^* mice at 14 and 28 dpi. Data are presented as means ± SD. Statistical significance was determined using one-way ANOVA with Tukey’s multiple comparisons test and Student’s *t* test.

Then, a RCRT model in wild-type controls and *Nlrp3^−/−^* mice was established to evaluate the regeneration of the enthesis (Fig. 2A). H&E and toluidine blue staining revealed more extensive fibrocartilage regeneration in *Nlrp3^−/−^* mice at 14 and 28 dpi (Fig. 2, B and C). Consistent with the histological staining, the modified tendon maturing score indicated that the regeneration of the enthesis in *Nlrp3^−/−^* mice was superior to that of wild-type controls (Fig. 2D). Bone regeneration of the enthesis was assessed by micro–computed tomography (micro-CT). Quantitative analysis of BMD, BV/TV, Tb.N, bone surface/bone volume (BS/BV), trabecular thickness (Tb.Th), and trabecular separation (Tb.Sp) revealed that bone regeneration of the enthesis in *Nlrp3^−/−^* mice was more significant than that in wild-type controls at 14 and 28 dpi (Fig. 2, E to H, and fig. S5, A to C). The expression of Tnmd and collagen II was used to evaluate the regeneration of tendon and fibrocartilage. IHC staining and IOD quantitative analysis of Tnmd and collagen II expression revealed superior tendon and fibrocartilage regeneration in *Nlrp3^−/−^* mice compared to wild-type controls (fig. S5, D to G). Furthermore, the functional recovery of the enthesis was evaluated via biomechanical tests. The failure load, stiffness, and modulus of the enthesis in *Nlrp3^−/−^* mice were considerably higher than that in wild-type controls at 28 dpi (Fig. 2, I to K, and fig. S5J). Other biomechanical parameters exhibited no substantial difference between *Nlrp3^−/−^* mice and wild-type controls (Fig. 2L and fig. S5, H, I, and K). The collective data indicate that the activation of NLRP3 inflammasomes suppresses enthesis regeneration.

### scRNA-seq uncovers that NLRP3 inflammasomes deteriorate inflammation and IL-1β inflammatory cross-talk

**Fig. 3.**
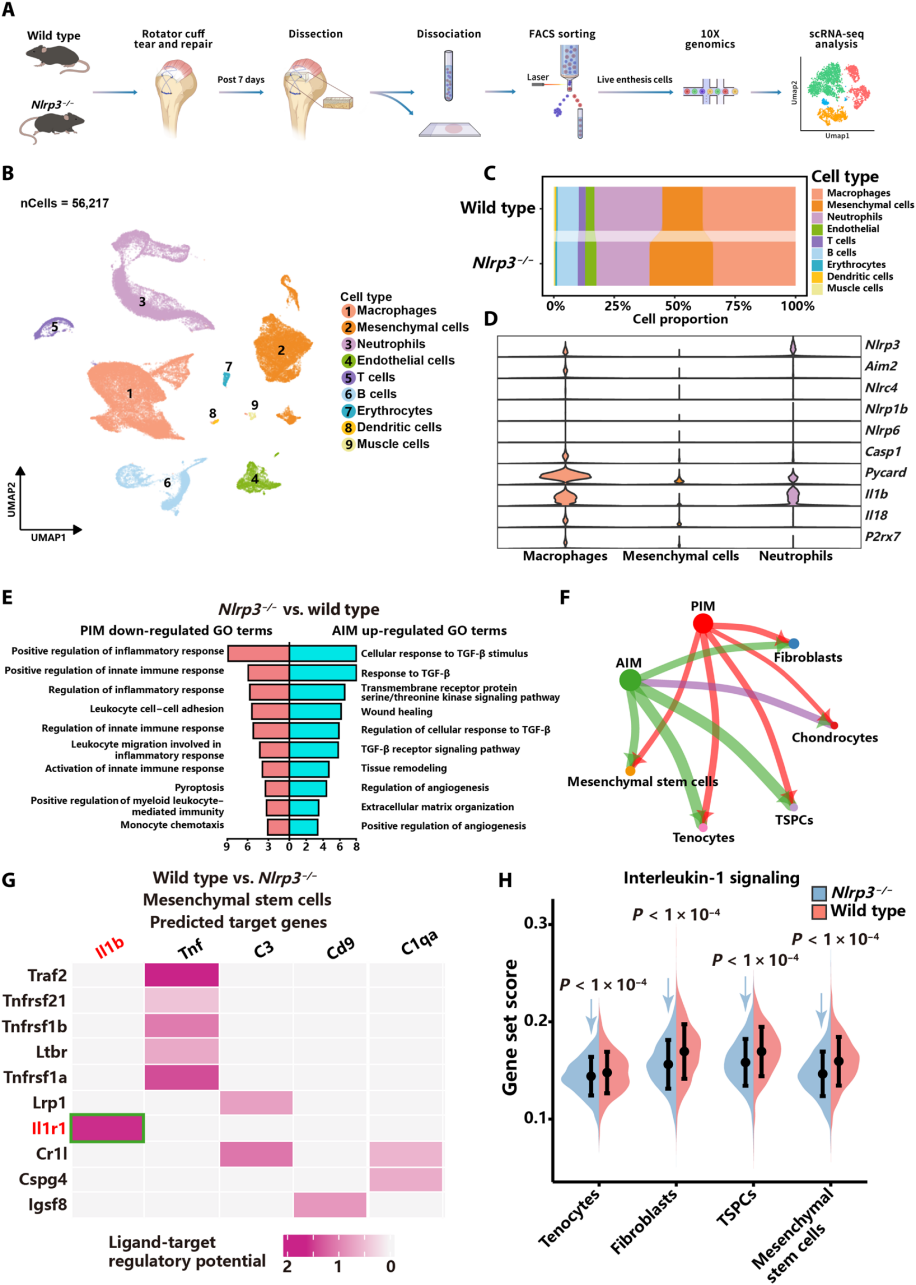
scRNA-seq uncovers that NLRP3 inflammasomes deteriorate inflammation and IL-1β inflammatory cross-talk. (**A**) Schematic of scRNA-seq, in which the enthesis was harvested from wild-type controls and *Nlrp3^−/−^* mice at 7 dpi and processed for scRNA-seq. (**B**) UMAP plot of 56,217 cells from wild-type controls (*n* = 3) and *Nlrp3^−/−^* mice (*n* = 3). (**C**) Bar plot of the proportions of nine major cell clusters in the injured enthesis of wild-type controls and *Nlrp3^−/−^* mice at 7 dpi. (**D**) Violin plots of specific gene expressions in macrophages, mesenchymal cells, and neutrophils. (**E**) GO enrichment analysis of down-regulated genes in PIM and up-regulated genes in AIM in *Nlrp3^−/−^* mice. (**F**) Circle plot of the interactions of subsets of macrophages and mesenchymal cells. Edge line thickness suggests the interaction strength between different cell clusters. (**G**) NicheNet analysis of ligand-target regulatory potential between macrophages and mesenchymal stem cells. (**H**) Gene set cores of IL-1 signaling in different mesenchymal cell subsets in wild-type controls and *Nlrp3^−/−^* mice. Statistical significance was determined using Student’s *t* test. FACS, fluorescence-activated cell sorting; TGF-β, transforming growth factor–β.

To unravel the mechanisms by which NLRP3 inflammasomes suppress enthesis regeneration, enthesis was harvested from wild-type controls and *Nlrp3^−/−^* mice at 7 dpi for scRNA-seq (Fig. 3A). This specific time point was selected for scRNA-seq as it represents the crucial shift from inflammatory to proliferative phase in enthesis regeneration.

**Fig. 4.**
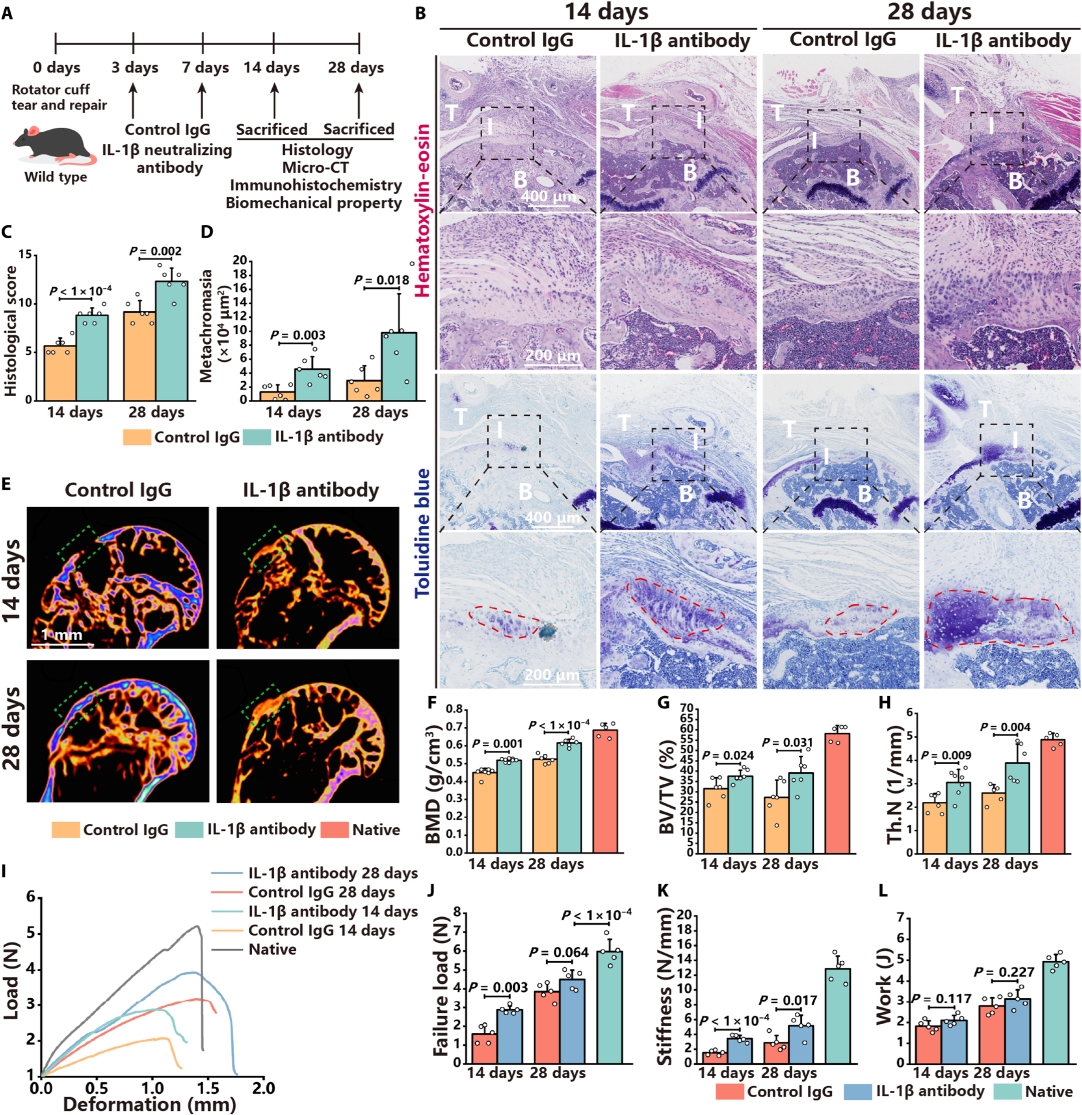
Blocking IL-1β inflammatory cross-talk with neutralizing antibodies accelerates enthesis regeneration. (**A**) Schematic of animal experiments, in which the RCTR model was established, and IL-1β neutralizing antibodies or control IgG was injected into the articular cavity, with analyses at 14 and 28 dpi. (**B**) H&E and toluidine blue staining of the enthesis in mice with IL-1β neutralizing antibodies or control IgG injection at 14 and 28 dpi. Black dashed squares represent the enlarged images of the enthesis. (**C** and **D**) Histological scores and metachromasia area size of the enthesis in mice with IL-1β neutralizing antibodies or control IgG injection at 14 and 28 dpi. (**E**) Micro-CT coronal views of the humerus in mice with IL-1β neutralizing antibodies or control IgG injection at 14 and 28 dpi. Green dashed squares represent the area of the enthesis. (**F** to **H**) Quantitative analysis of BMD, BV/TV, and Tb. N of the enthesis in mice with IL-1β neutralizing antibodies or control IgG injection and sham operation. (**I**) Deformation and load curves of the enthesis in mice with IL-1β neutralizing antibodies or control IgG injection at 14 and 28 dpi. (**J** to **L**) Failure load, stiffness, and work of the enthesis in mice with IL-1β neutralizing antibodies or control IgG injection at 14 and 28 dpi. Data are presented as means ± SD. Statistical significance was determined using one-way ANOVA with Tukey’s multiple comparisons test and Student’s *t* test.

Unbiased clustering identified nine clusters visualized with Uniform Manifold Approximation and Projection (UMAP) (Fig. 3B). A total of nine cell types were identified with cell type–specific markers, including macrophages (*Cd68*, *Csf1r*, and *Cd14*), neutrophils (*Lcn2*, *G0s2*, and *S100a8*), T cells (*Cd3d*, *Cd3g*, and *Ccl5*), erythrocytes (*Gata1*, *Hbb-bt*, and *Hbb-bs*), dendritic cells (*Siglech*, *Cd300c*, and *Ccr9*), B cells (*Cd79a*, *Cd79b*, and *Mzb1*), endothelial cells (*Vwf*, *Cd34*, and *Flt1*), muscle cells (*Myod1*, *Myf5*, and *Fgfr4*), and mesenchymal cells (*Col5a2*, *Col5a1*, and *Col6a1*) (Fig. 4B and fig. S6, A and B). Macrophages, neutrophils, and mesenchymal cells were the three most prevalent cell types in the injured enthesis at 7 dpi (Fig. 3C). In *Nlrp3^−/−^* mice, the proportions of macrophages and neutrophils decreased compared to wild-type controls, whereas the proportion of mesenchymal cells increased (Fig. 3C). There were no obvious differences in the proportions of other cell types between wild-type controls and *Nlrp3^−/−^* mice (Fig. 3C). Genes related to NLRP3 inflammasomes (*Nlrp3*, *Casp1*, *Pycard*, *Il1b*, and *Il18*) were primarily expressed in macrophages (Fig. 3D). In addition, the assessment of other types of inflammasomes indicated that *Aim2* was predominantly expressed in macrophages, while the expression levels of *Nlrc4*, *Nlrp1b*, and *Nlrp6* were negligible (Fig. 3D).

Feature plots showed that a subset of macrophages in wild-type controls coexpressed *Nlrp3* and *Il1b* (fig. S7A). Despite the negligible expression of *Nlrp3* in *Nlrp3^−/−^* mice, *Il1b*-positive macrophages subset in *Nlrp3^−/−^* mice had similar gene expression profiles with wild-type controls (fig. S7B). Since *Il1b*-positive macrophages also exhibited elevated expression of additional proinflammatory genes (*Il6*, *Ptgs2*, and *Ly6c2*), this specific subset of macrophages was classified as PIM (fig. S7, C and D). Another subset with increased anti-inflammatory and canonical M2 marker genes (*Ccl8*, *C1qa*, *Mrc1*, and *Arg1*) was named anti-inflammatory macrophages (AIM) (fig. S7, C and D). Another subset of macrophages was classified as osteoclasts based on marker genes (*Acp5*, *Atp6v0d2*, *Ctsk*, and *Mmp9*) (fig. S7, C and D). The proportion of PIM was decreased in *Nlrp3^−/−^* mice compared to wild-type controls, whereas the proportion of AIM exhibited an opposite trend (fig. S7E). Comparing DEGs in PIM revealed that genes promoting inflammation (*Nlrp3*, *Ninj1*, *S100a9*, *Zbp1*, and *Aif1*) exhibited lower expression in *Nlrp3^−/−^* mice (fig. S7F). GO analysis of down-regulated genes in PIM revealed that biological processes including pyroptosis, positive regulation of inflammatory response, positive regulation of innate immune response, and leukocyte migration involved in inflammatory response were suppressed in *Nlrp3^−/−^* mice, indicating that the inflammation was attenuated in *Nlrp3^−/−^* mice (Fig. 3E). DEGs in AIM exhibited elevated expression of genes associated with inflammation resolution (*Gas6*, *Gdf3*, *Sulf2*, *Fcgr2b*, and *Tgfbr2*) in *Nlrp3^−/−^* mice (fig. S7G). The analysis of up-regulated genes in AIM suggested that biological processes including response to transforming growth factor–β, wound healing, regulation of angiogenesis, and tissue remodeling, which are closely related to inflammation resolution and regeneration, were up-regulated in *Nlrp3^−/−^* mice (Fig. 3E). These data suggest that *Nlrp3* KO ameliorates inflammation, accelerates inflammation resolution, and facilitates regeneration by regulating functions of macrophages.

To further evaluate the influence of NLRP3 inflammasomes on enthesis regeneration, mesenchymal cells were subdivided into nine subsets based on marker genes: tenocytes (*Tnmd*, *Fomd*, and *Thbs4*), fibroblasts (*Mfap4*, *Mest*, and *Fth1*), tendon stem/progenitor cells (TSPCs; *Tnc*, *Scx*, and *Tagln*), mesenchymal stem cells (*Cebpd*, *Ly6a*, and *Pdgfra*), chondrocytes (*Col2a1*, *Sox9*, and *Col10a1*), adipocytes (*Lpl*, *Cyp7b1*, and *Creb3l3*), myofibroblasts (*Ppp1r14a*, *Myh11*, and *Parm1*), synovial cells (*Prg4*, *Has1*, and *Htr2a*), and osteocytes (*Bglap*, *Col1a1*, and *Dmp1*) (fig. S8, A and B). The proportions of tenocytes, TSPCs, and chondrocytes increased in *Nlrp3^−/−^* mice (fig. S8C). To validate scRNA-seq results, immunofluorescence staining of CD68^+^ macrophages and Pdgfrα^+^ stem cells indicated that the number of stem cells was higher in *Nlrp3^−/−^* mice than wild-type controls (fig. S8, D and E). While the quantity of macrophages was reversed (fig. S8F), up-regulated genes of tenocytes in *Nlrp3^−/−^* mice are primarily enriched in the following biological processes: extracellular matrix organization, positive regulation of angiogenesis, tissue regeneration, and transforming growth factor–β receptor signaling (fig. S9A). GO analysis of mesenchymal stem cells in *Nlrp3^−/−^* mice showed enrichment of up-regulated pathways related to transforming growth factor, mesenchymal cell differentiation, and tissue regeneration (fig. S9A). Enrichment of up-regulated genes in chondrocytes in *Nlrp3^−/−^* mice was primarily involved in cartilage development, chondrocyte differentiation, extracellular matrix organization, and chondrocyte development (fig. S9A). The above results indicate that *Nlrp3* KO improves the regenerative capacities of tenocytes, mesenchymal stem cells, and chondrocytes.

Since *Nlrp3* was primarily expressed in macrophages and was almost absent in mesenchymal stem cells, it was hypothesized that the NLRP3 inflammasomes modulated enthesis regeneration and repair through cross-talk between macrophages and mesenchymal stem cells. A chord plot revealed frequent interactions between macrophages and mesenchymal stem cells (Fig. 3F). Considering that IL-1β was the dominant product of NLRP3 inflammasomes, the expression of the IL-1β receptor, IL-1 receptor type 1 (IL1R1), was further analyzed. *Il1r1* was expressed in mesenchymal stem cells, indicating that cross-talk between macrophages and mesenchymal cells might be mediated by IL-1β (fig. S10, A and B). NicheNet analysis demonstrated a stronger interaction of *ll1b-Il1r1* between macrophages and mesenchymal stem cells in wild-type controls compared to *Nlrp3^−/−^* mice (Fig. 3G). Moreover, gene set scores of IL-1β signaling in TSPCs and mesenchymal stem cells were lower in *Nlrp3^−/−^* mice, suggesting that the effect of IL-1β signaling on mesenchymal stem cells was attenuated (Fig. 3H). These results suggest that NLRP3 inflammasomes modulate IL-1β inflammatory cross-talk between macrophages and stem cells.

Since previous results showed that the proportion of AIM was higher in *Nlrp3^−/−^* mice, the gene set scores of anti-inflammatory pathways in AIM and PIM were also evaluated. Unexpectedly, gene set scores of IL-10 signaling and IL-4 and IL-13 signaling were higher in both AIM and PIM in *Nlrp3^−/−^* mice, indicating that *Nlrp3* KO promoted the polarization of macrophages toward the anti-inflammatory phenotype and accelerated inflammation resolution (fig. S10, C and D). Together, scRNA-seq depicts that NLRP3 inflammasomes exacerbate inflammation and IL-1β inflammatory cross-talk between macrophages and mesenchymal stem cells.

### Blocking IL-1β inflammatory cross-talk with neutralizing antibodies accelerates enthesis regeneration

scRNA-seq suggested that cross-talk between macrophages and mesenchymal stem cells might be mediated by IL-1β and that blocking NLRP3 inflammasomes attenuated the IL-1β signaling pathway in mesenchymal stem cells. Thus, it was speculated that IL-1β inflammatory cross-talk contributed to poor enthesis regeneration. This hypothesis was tested by intra-articular injection of IL-1β neutralizing antibodies. First, the retention period of neutralizing antibodies in the articular cavity was measured. In vivo imaging demonstrated that neutralizing antibodies remained in the articular cavity for up to 72 hours after injection (fig. S11, A and B). Considering the trend of IL-1β concentration and retention period of neutralizing antibodies, IL-1β neutralizing antibodies and control immunoglobulin G (IgG) were injected into the articular cavity at 3 and 7 dpi, and the regeneration of the enthesis was evaluated at 14 and 28 dpi (Fig. 4A).

Mice injected with IL-1β neutralizing antibodies had better enthesis continuity and higher histological scores than those injected with control IgG (Fig. 4, B and C). In addition, a larger metachromasia area was observed in mice injected with IL-1β neutralizing antibodies, indicating that the inhibition of IL-1β signaling accelerates enthesis regeneration (Fig. 4, B and D). Quantitative analysis revealed that mice injected with IL-1β neutralizing antibodies exhibited greater bone mass at the enthesis than those injected with control IgG (Fig. 4, E to H, and fig. S12, A to C). Moreover, IHC staining and IOD quantitative analysis of Tnmd and collagen II revealed that IL-1β neutralizing antibodies facilitated tendon and fibrocartilage regeneration of the enthesis (fig. S12, D to G). The enthesis failure load, stiffness, ultimate stress, and toughness were significantly higher in mice injected with IL-1β neutralizing antibodies than those injected with control IgG (Fig. 4, I to L, and fig. S12, H and K). Overall, blocking IL-1β signaling with neutralizing antibodies accelerates enthesis regeneration.

### NLRP3 inflammasomes suppress the secretion of anti-inflammatory cytokines by macrophages to inhibit inflammation resolution

scRNA-seq revealed that stem cells received fewer proinflammatory signals, while macrophages received more anti-inflammatory signals in *Nlrp3^−/−^* mice. Since NLRP3 inflammasomes were primarily expressed in macrophages and macrophages were the major source of various inflammatory cytokines, it was hypothesized that NLRP3 inflammasomes modulated the secretion of proinflammatory and anti-inflammatory cytokines by macrophages to regulate the injured enthesis niche.

**Fig. 5.**
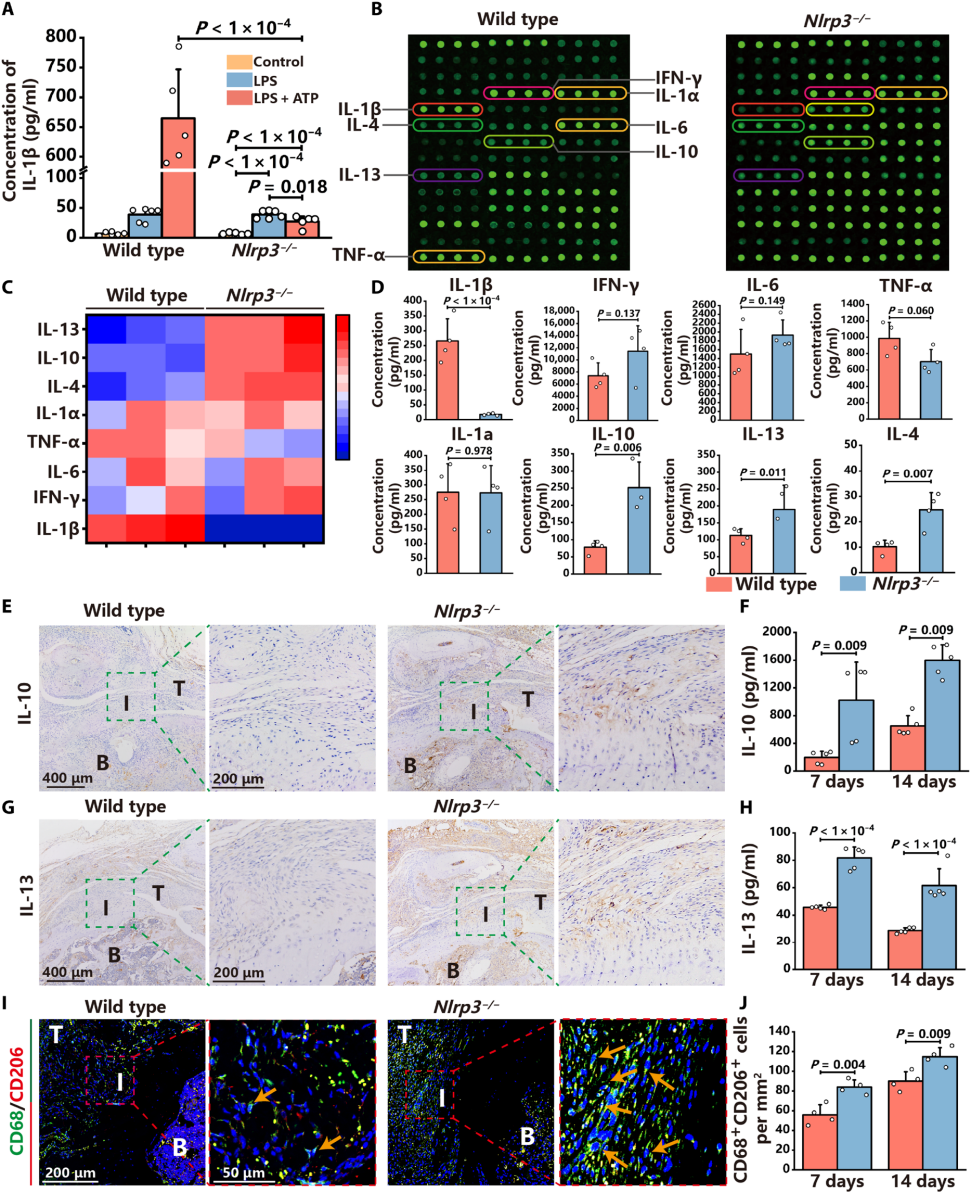
NLRP3 inflammasomes suppress the secretion of anti-inflammatory cytokines by macrophages to inhibit inflammation resolution. (**A**) IL-1β concentration in the supernatant of wild-type and *Nlrp3^−/−^* BMDMs. (**B**) Scan images of the supernatant of wild-type and *Nlrp3^−/−^* BMDMs in the mouse inflammation array Q1. (**C**) Heatmap of inflammation factors in the supernatant of wild-type and *Nlrp3^−/−^* BMDMs. (**D**) Concentrations of IL-1β, IFN-γ, IL-6, TNF-α, IL-1α, IL-10, IL-13, and IL-4 in the supernatant of wild-type and *Nlrp3^−/−^* BMDMs. (**E** to **H**) IHC staining and ELISA analysis of IL-10 and IL-13 in the enthesis of wild-type and *Nlrp3^−/−^* mice. Green dashed squares represent the enlarged images of the enthesis. (**I** and **J**) Immunofluorescence staining and quantification of CD68 (green)– and CD206 (red)–positive cells in the enthesis of wild-type controls and *Nlrp3^−/−^* mice. Red dashed squares represent the enlarged images of the enthesis. Arrows indicate CD68^+^ and CD206^+^ macrophages. Data are presented as means ± SD. Statistical significance was determined using one-way ANOVA with Tukey’s multiple comparisons test and Student’s *t* test.

It has been indicated that costimulation with lipopolysaccharide (LPS) and adenosine triphosphate (ATP) activates NLRP3 inflammasomes in bone marrow–derived macrophages (BMDMs) (*27*). Here, LPS and ATP costimulation increased the production of IL-1β in wild-type BMDMs (Fig. 5A). In contrast, this effect was diminished in *Nlrp3^−/−^* BMDMs (Fig. 5A). Furthermore, a mouse inflammation array Q1 was carried out to quantify various inflammatory cytokines in the supernatant of wild-type BMDMs and *Nlrp3^−/−^* BMDMs after LPS and ATP costimulation. Scan images, heatmap, and quantitative analysis indicated that IL-1β levels in the *Nlrp3^−/−^* BMDMs supernatant were much lower than wild-type controls (Fig. 5, B to D). Nevertheless, the concentrations of other important proinflammatory cytokines, including IL-6, interferon-γ (IFN-γ), tumor necrosis factor–α (TNF-α), and IL-1α, did not exhibit any statistical difference (Fig. 5, B to D). In addition, it was observed that the concentrations of anti-inflammatory cytokines, including IL-4, IL-10, and IL-13, were significantly increased in the supernatant of *Nlrp3^−/−^* BMDMs compared to wild-type BMDMs (Fig. 5, B to D).

Then, the in vivo regulatory effect of NLRP3 inflammasomes on anti-inflammatory cytokines and inflammation resolution was investigated. There was neither IL-10 nor IL-13 expression in native enthesis (fig. S13, A and B). Furthermore, IHC staining analysis revealed that IL-10– and IL-13–positive areas were larger in the enthesis of *Nlrp3^−/−^* mice than wild-type controls at 14 dpi (Fig. 5, E and G). The concentrations of IL-10 and IL-13 were significantly higher in the injured enthesis of *Nlrp3^−/−^* mice than in wild-type controls at 7 and 14 dpi (Fig. 5, F and H). The amount of CD68^+^ and CD206^+^ AIM was significantly higher in the injured enthesis of *Nlrp3^−/−^* mice at 7 and 14 dpi (Fig. 5, I and J). Collectively, these data indicate that NLRP3 inflammasomes not only enhance the production of IL-1β but also inhibit the secretion of anti-inflammatory cytokines, resulting in prolonged and uncontrolled inflammation that hinders enthesis regeneration.

### NLRP3 inflammasomes inhibit the production of docosatrienoic acid

**Fig. 6.**
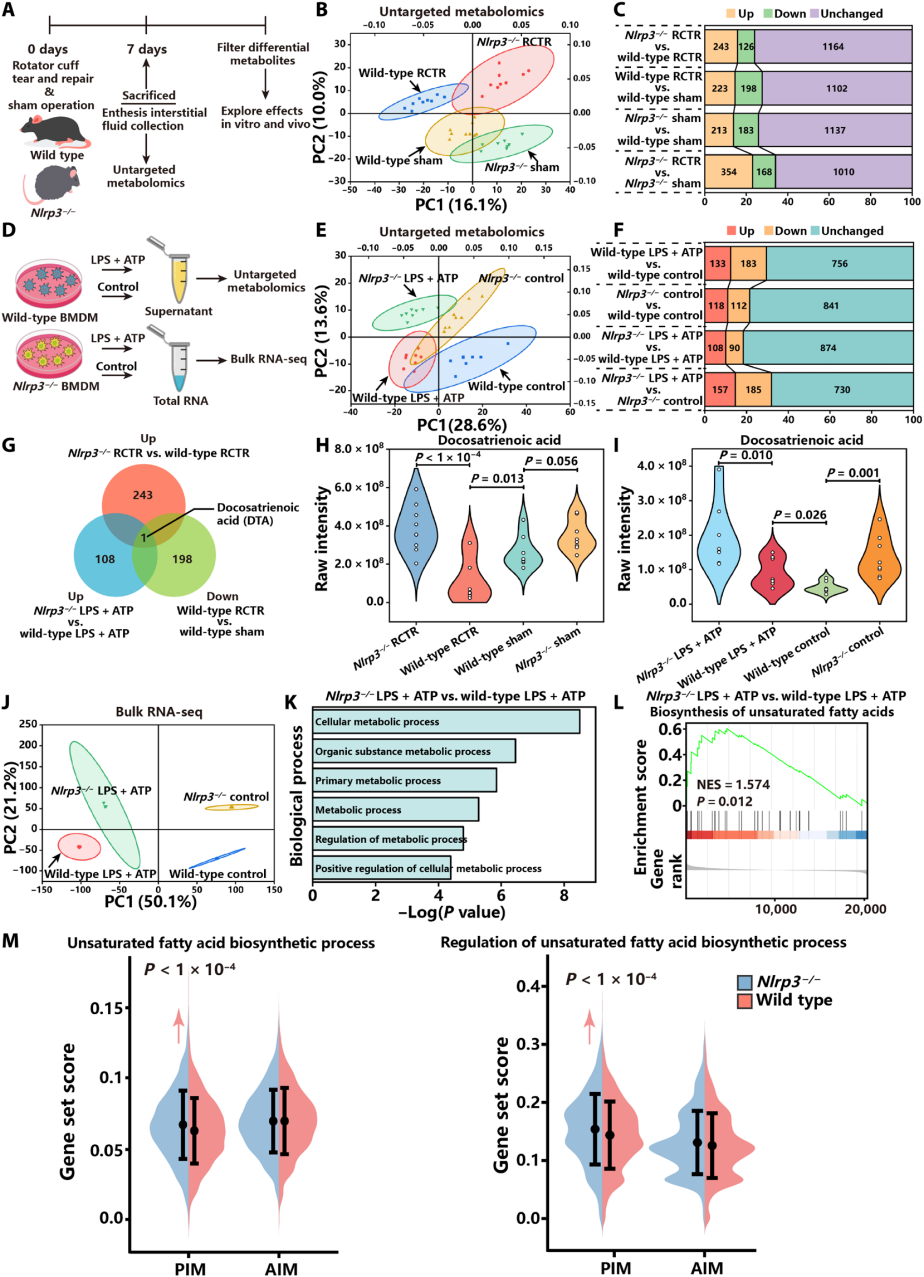
NLRP3 inflammasomes inhibit the production of docosatrienoic acid. (**A**) Schematic of untargeted metabolomics of EIF. (**B**) PCA of untargeted metabolomics of EIF. (**C**) The number of differential metabolites in EIF. (**D**) Schematic of untargeted metabolomics and RNA sequencing of wild-type and *Nlrp3^−/−^* BMDMs. (**E**) PCA of untargeted metabolomics of supernatant from wild-type and *Nlrp3^−/−^* BMDMs. (**F**) The number of differential metabolites in the supernatant. (**G**) Venn diagram showing significantly enriched metabolites in different experimental setting. (**H** and **I**) Mass spectrometer quantification of DTA in EIF and supernatant. (**J**) PCA of RNA sequencing of wild-type and *Nlrp3^−/−^* BMDMs. (**K**) GO enrichment analysis of differentially expressed pathways of BMDMs. (**L**) Gene set enrichment analysis (GSEA) of biosynthesis of unsaturated fatty acids. (**M**) Gene set cores of unsaturated fatty acid biosynthetic process and regulation of unsaturated fatty acid biosynthetic process in PIM and AIM in wild-type controls and *Nlrp3^−/−^* mice. Statistical significance was determined using one-way ANOVA with Tukey’s multiple comparisons test and Student’s *t* test.

Considering that NLRP3 inflammasomes alter macrophage metabolic processes and thus regulate metabolite production, we suspect that NLRP3 inflammasome–mediated metabolic reprogramming may also modulate enthesis regeneration. To investigate metabolite profile changes in the enthesis niche induced by NLRP3 inflammasome, the enthesis interstitial fluid (EIF) from wild-type controls and *Nlrp3^−/−^* mice with injury or sham operation was extracted for untargeted metabolomics (Fig. 6A). PCA revealed that EIF metabolite profiles between wild-type controls and *Nlrp3^−/−^* mice are distinctly different at 7 dpi or with sham operation (Fig. 6B). There were 243 up-regulated metabolites and 126 down-regulated metabolites in *Nlrp3^−/−^* mice compared with wild-type controls at 7 dpi (Fig. 6C). To further explore the NLRP3 inflammasome–triggered metabolic and transcriptomic changes in macrophages, we collected the supernatant for untargeted metabolomics and extracted total RNA for RNA-seq from wild-type and *Nlrp3^−/−^* BMDMs, both with or without stimulation (Fig. 6D). Regardless of stimulation, there were substantial disparities between the supernatants from wild-type and *Nlrp3^−/−^* BMDMs (Fig. 6E). Compared to wild-type BMDMs, there were 108 up-regulated metabolites and 90 down-regulated metabolites in the supernatant of *Nlrp3^−/−^* BMDMs (Fig. 6F). After analyzing the differential metabolites in vivo and in vitro, it was discovered that DTA was consistently enriched in EIF of *Nlrp3^−/−^* mice at 7 dpi and the supernatant of *Nlrp3^−/−^* BMDMs with stimulation (Fig. 6, G to I), while the abundance of DTA in EIF was significantly reduced in wild-type mice at 7 dpi in contrast with sham operation (Fig. 6H).

DTA is a kind of polyunsaturated fatty acid (PUFAs). Although the specific metabolic process of DTA in macrophages is still unclear, some literature has suggested that DTA may be synthesized from arachidonic acid and other ω-6–derived PUFAs (*28*). According to RNA-seq, DEGs of *Nlrp3^−/−^* BMDMs were enriched in GO terms related to metabolic processes (Fig. 6, J and K). Gene set enrichment analysis (GSEA) indicated that biosynthesis of unsaturated fatty acids pathway was up-regulated in *Nlrp3^−/−^* BMDMs with stimulation (Fig. 6L). Regardless of activation status, the differential metabolites and DEGs in *Nlrp3^−/−^* BMDMs were enriched in metabolic processes related to PUFA synthesis, including glycerophospholipid, arachidonic acid, and glycerolipid metabolism, as well as the biosynthesis of unsaturated fatty acids (fig. S14A). scRNA-seq demonstrated that gene set scores of unsaturated fatty acid biosynthetic process and its regulation in PIM were significantly higher in *Nlrp3^−/−^* mice than wild-type controls (Fig. 6M). These findings demonstrate that NLRP3 inflammasomes reprogram metabolic processes and inhibit DTA synthesis in vitro and in vivo.

### Docosatrienoic acid boosts cell proliferation and enthesis regeneration

**Fig. 7.**
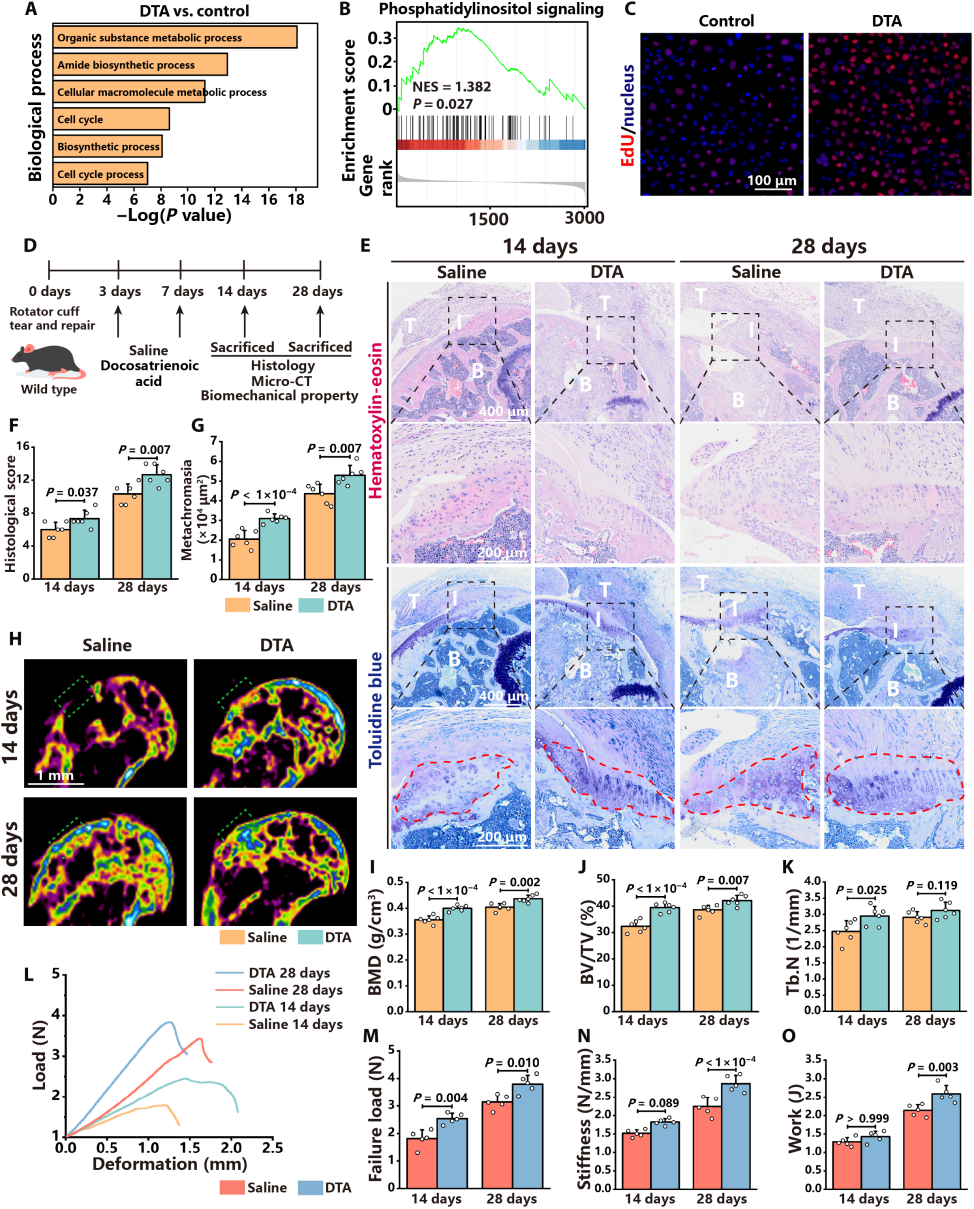
Docosatrienoic acid boosts cell proliferation and enthesis regeneration. (**A**) GO enrichment analysis of differentially expressed pathways of BMSCs. (**B**) GSEA of phosphatidylinositol signaling. NES, normalized enrichment score. (**C**) EdU (red) and nucleus (blue) staining of BMSCs with or without DTA treatment. (**D**) Schematic of animal experiments, in which DTA was replenished at 3 and 7 dpi. (**E**) H&E and toluidine blue staining of the enthesis in mice with DTA supplement or saline injection at 14 and 28 dpi. Black dashed squares represent the enlarged images of the enthesis. (**F** and **G**) Histological scores and metachromasia area size of the enthesis in mice with DTA supplement or saline injection at 14 and 28 dpi. (**H**) Micro-CT coronal views of the humerus of mice with DTA supplement or saline injection at 14 and 28 dpi. Green dashed squares represent the area of the enthesis. (**I** to **K**) Quantitative analysis of BMD, BV/TV, and Tb.N of the enthesis. (**L**) Deformation and load curves of the enthesis in mice with DTA supplement or saline injection at 14 and 28 dpi. (**M** to **O**) Failure load, stiffness, and work of the enthesis in mice with DTA supplement or saline injection at 14 and 28 dpi. Data are presented as means ± SD. Statistical significance was determined using one-way ANOVA with Tukey’s multiple comparisons test and Student’s *t* test.

The effects of DTA on stem cells and enthesis regeneration were further investigated. Bone marrow–derived stem cells (BMSCs) were treated with 50 μM DTA for RNA-seq. DTA treatment substantially changed the gene profiles of BMSCs (fig. S15A). DEGs were enriched in GO terms related to metabolic processes, biosynthetic processes, and cell cycles (Fig. 7A). GSEA indicated that phosphatidylinositol signaling was up-regulated in BMSCs after DTA treatment (Fig. 7B). 5-Ethynyl-2′-deoxyuridine (EdU) assay demonstrated that DTA promoted BMSC proliferation (Fig. 7C and fig. S15B). Western blot suggested that phosphatidylinositol signaling was activated in BMSCs after DTA treatment (fig. S15C). In addition, gene set scores of mesenchymal stem cell proliferation and phosphatidylinositol signaling were higher in mesenchymal stem cell in *Nlrp3^−/−^* mice (fig. S15D).

Mice with DTA supplements exhibited superior histological scores and improved enthesis continuity (Fig. 7, D to F). Replenishing DTA increased the area of metachromasia compared to saline, indicating that DTA promoted fibrocartilage regeneration (Fig. 7G). Micro-CT and quantitative analysis revealed that mice supplemented with DTA exhibited greater bone mass at the enthesis than those with saline (Fig. 7, H to K). Biomechanical tests revealed that replenishing DTA facilitated the functional recovery of enthesis (Fig. 7, L to O). Together, DTA promotes the proliferation of stem cells and boosts enthesis regeneration.

### NLRP3 inflammasome–mediated secretome suppresses the differentiation and migration of stem cells

Previous experiments illustrated that NLRP3 inflammasomes regulate not only the secretion of inflammatory cytokines but also the production of metabolites. To further investigate the general effects of secretome modulated by NLRP3 inflammasomes on stem cells, the conditional medium (CM) of wild-type BMDMs and *Nlrp3^−/−^* BMDMs was collected to treat stem cells. Alizarin red S (ARS) and alkaline phosphatase (ALP) staining demonstrated that both wild-type and *Nlrp3^−/−^* CM reduced the cellular mineralization and ALP activity of BMSCs, while the inhibitory effect of *Nlrp3^−/−^* CM was ameliorated (fig. S16A). The expression of osteogenesis-related genes including *Col1a1*, *OCN*, and *Runx2* also indicated that the inhibitory effect of *Nlrp3^−/−^* CM on BMSCs was reduced (fig. S16, B to D). The effect of wild-type and *Nlrp3^−/−^* CM on chondrogenic capabilities of BMSCs was also investigated. Alcian blue staining, Safranin O staining, collagen II IHC, Bern score, and volume of cartilage pellets indicated that both wild-type and *Nlrp3^−/−^* CM significantly reduced the synthesis of the extracellular matrix; however, these effects were attenuated in *Nlrp3^−/−^* CM (fig. S16, E to G). The expression of chondrogenic marker genes in BMSCs cultured with *Nlrp3^−/−^* CM was significantly higher than that in BMSCs cultured with wild-type CM (fig. S16, H to J).

The impact of wild-type and *Nlrp3^−/−^* CM on the tenogenic differentiation of TSPCs was also assessed. Sirius red staining indicated that wild-type CM notably reduced the synthesis of the extracellular matrix in TSPCs, and this effect was ameliorated in *Nlrp3^−/−^* CM (fig. S16K). The transcription levels of tenogenic differentiation marker genes *Tnmd*, *Scx*, and *Mkx* in TSPCs cultured with *Nlrp3^−/−^* CM were significantly higher than that in TSPCs cultured with wild-type CM (fig. S16, L to N). Moreover, the transwell assay showed that both wild-type and *Nlrp3^−/−^* CM inhibited stem cell migration, but this effect was attenuated in *Nlrp3^−/−^* CM (fig. S16, O and P). Together, these data suggest that NLRP3 inflammasome–mediated secretome suppresses the differentiation and migration of stem cells.

### Conditional KO of *P2rx7* in myeloid cells reduces NLRP3 inflammasome activity after enthesis injury and improves enthesis regeneration

**Fig. 8.**
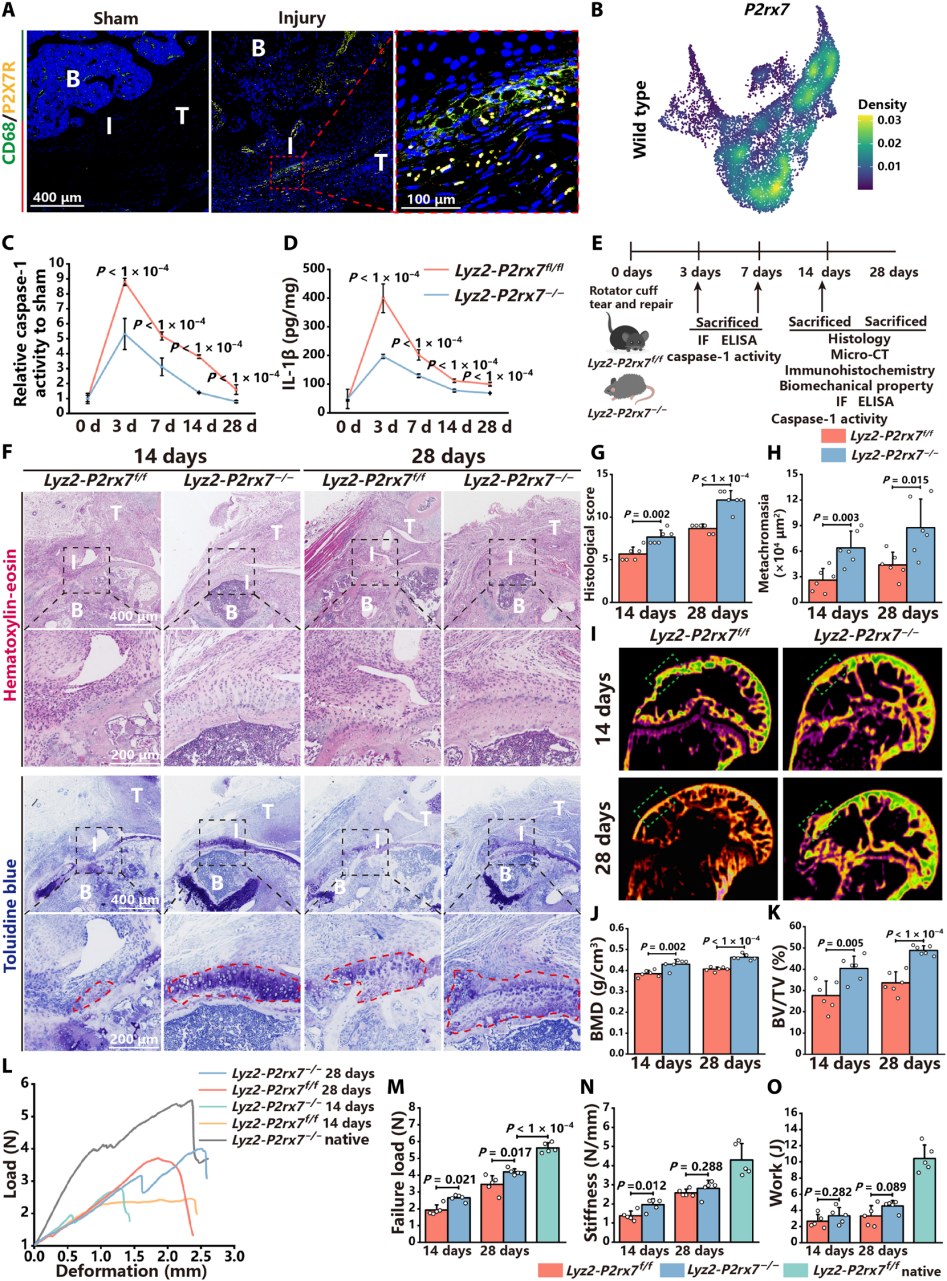
Conditional KO of *P2rx7* in myeloid cells reduces NLRP3 inflammasome activation after enthesis injury and improves enthesis regeneration. (**A**) Immunofluorescence staining of CD68 (green) and P2X7R (yellow) in native and injured enthesis. Red dashed squares represent enlarged images of the enthesis. (**B**) Feature plots of single-cell gene expression of *P2rx7* in macrophages in wild-type mice. (**C** and **D**) The relative caspase-1 activity and concentration of IL-1β in the enthesis of *Lyz2-P2rx7^f/f^* and *Lyz2-P2rx7^−/−^* mice at 3, 7, 14, and 28 dpi. (**E**) Schematic of animal experiments, in which the RCTR model was established in *Lyz2-P2rx7^f/f^* and *Lyz2-P2rx7^−/−^* mice, and analyzed at 3, 7, 14, and 28 dpi. (**F**) H&E and toluidine blue staining of the enthesis in *Lyz2-P2rx7^f/f^* and *Lyz2-P2rx7^−/−^* mice at 14 and 28 dpi. Black dashed squares represent enlarged images of the enthesis. (**G** and **H**) Histological scores and the metachromasia area size of the enthesis in *Lyz2-P2rx7^f/f^* and *Lyz2-P2rx7^−/−^* mice at 14 and 28 dpi. (**I**) Micro-CT coronal views of the humerus of *Lyz2-P2rx7^f/f^* and *Lyz2-P2rx7^−/−^* mice at 14 and 28 dpi. Green dashed squares represent the area of interest. (**J** and **K**) Quantitative analysis of the BMD and BV/TV of the enthesis. (**L**) Deformation and load curves of the enthesis in *Lyz2-P2rx7 ^f/f^* and *Lyz2-P2rx7^−/−^* mice at 14 and 28 dpi. (**M** to **O**) Failure load, stiffness, and work of the enthesis in mice with IL-1β neutralizing antibodies or control IgG injection at 14 and 28 dpi. Data are presented as means ± SD. Statistical significance was determined using one-way ANOVA with Tukey’s multiple comparisons test and Student’s *t* test.

This study revealed that the activation of NLRP3 inflammasomes inhibits enthesis regeneration. However, it remains unclear how enthesis injury activates NLRP3 inflammasomes. Previous literature reported that the ion channel P2X7R on cell membranes mediated potassium efflux, which induced the activation of NLRP3 inflammasomes (*20*). RNA-seq and RT-qPCR revealed that the transcriptional level of *P2rx7* was elevated after enthesis injury (Fig. 1, C and D). Furthermore, immunofluorescence staining and scRNA-seq showed that P2X7R was primarily expressed in macrophages (Fig. 8, A and B). Therefore, it was hypothesized that P2X7R might regulate the activation of NLRP3 inflammasomes after enthesis injury. To verify this hypothesis, *P2rx7* complete KO mice (*P2rx7* KO) were created at first. Unexpectedly, it was observed that *P2rx7* complete KO disrupted the arrangement of fibrocartilage cells (fig. S17, A and B). The bone mass and volume of enthesis in *P2rx7* KO mice were significantly reduced compared to wild-type controls (fig. S17, C to F). The aforementioned data suggest that P2X7R might influence the development of enthesis. Consequently, *Lyz2-Cre* mice were crossed with *P2rx7* ^*flox/flo*x^ mice to obtain *Lyz2-cre*:: *P2rx7*^*flox/flo*x^ mice (*Lyz2-P2rx7^−/−^*) with the conditional KO of *P2rx7* in myeloid cells. Histological staining indicated that the conditional KO of *P2rx7* in myeloid cells did not influence the structure of enthesis (fig. S17, G and H). Therefore, *Lyz2-P2rx7^−/−^* and *Lyz2-P2rx7^f/f^* mice were used for further experiments.

*Lyz2-P2rx7^f/f^* and *Lyz2-P2rx7^−/−^* BMDMs were costimulated with LPS and ATP to induce the activation of NLRP3 inflammasomes. IL-1β concentration in the supernatant of *Lyz2-P2rx7^f/f^* BMDMs was significantly higher than that of *Lyz2-P2rx7^−/−^* BMDMs, indicating the successful KO of *P2rx7* in macrophages (fig. S18A). The relative caspase-1 activity and IL-1β concentration of the enthesis peaked at 3 dpi in both *Lyz2-P2rx7^f/f^* and *Lyz2-P2rx7^−/−^* mice and gradually declined to baseline levels at 28 dpi (Fig. 8, C and D). The relative caspase-1 activity and IL-1β concentration of *Lyz2-P2rx7^−/−^* mice were significantly lower than those of *Lyz2-P2rx7^f/f^* mice at all time points postinjury (Fig. 8, C and D). Immunofluorescence staining revealed that the colocalization of NLRP3 and caspase-1 formed specks in macrophages at the enthesis of both *Lyz2-P2rx7^f/f^* and *Lyz2-P2rx7^−/−^* mice following injury; however, the number of specks per macrophage in *Lyz2-P2rx7^−/−^* mice was significantly less than *Lyz2-P2rx7^f/f^* mice (fig. S18, B and C). Overall, conditional KO of *P2rx7* in myeloid cells reduces NLRP3 inflammasome activity after enthesis injury.

The impact of P2X7R on the regeneration of the enthesis was further investigated (Fig. 8E). H&E and toluidine blue staining revealed improved continuity of the enthesis at both time points in *Lyz2-P2rx7^−/−^* mice compared to the *Lyz2-P2rx7^f/f^* controls (Fig. 8F). *Lyz2-P2rx7^−/−^* mice exhibited higher histological scores and larger areas of toluidine blue metachromasia than *Lyz2-P2rx7^f/f^* mice (Fig. 8, G and H). Moreover, quantitative analysis of micro-CT indicated that the bone mass of the enthesis in *Lyz2-P2rx7^−/−^* mice was substantially higher compared to that in *Lyz2-P2rx7^f/f^* mice (Fig. 8, I to K, and fig. S19, A to D). The IHC staining and IOD analysis revealed that *Lyz2-P2rx7^−/−^* mice had significantly higher expression levels of Tnmd and collagen II than *Lyz2-P2rx7^f/f^* mice (fig. S19, E to H). The results of biomechanical testing indicated that the functional recovery of the enthesis was considerably greater in *Lyz2-P2rx7^−/−^* mice at 14 and 28 dpi than in *Lyz2-P2rx7^f/f^* mice (Fig. 8, L to O, and fig. S19, I and L). Together, the above results suggest that the conditional KO of *P2rx7* in myeloid cells reduces NLRP3 inflammasome activity after enthesis injury and promotes enthesis regeneration.

## DISCUSSION

**Fig. 9.**
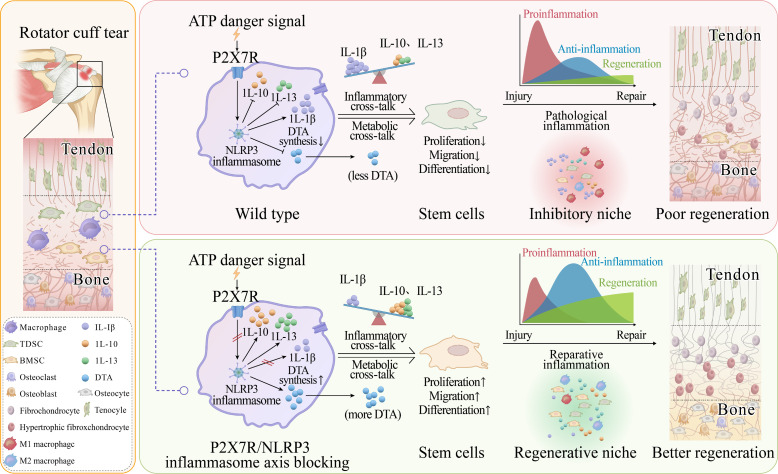
The schematic of this study. After enthesis injury, NLRP3 inflammasomes are activated in infiltrated macrophages upon receiving activation signals mediated by P2X7R. The activation of the P2X7R/NLRP3 inflammasome axis not only exacerbates inflammation by prompting the release of IL-1β and suppressing the production of anti-inflammatory factors including IL-10 and IL-13 but also inhibits the production of proregenerative docosatrienoic acid. NLRP3 inflammasomes suppress enthesis regeneration via aggravating IL-1β inflammatory cross-talk and restraining docosatrienoic acid metabolic cross-talk between macrophages and stem cells. Blocking the P2X7R/NLRP3 inflammasome axis rewires the cross-talk between macrophages and stem cells and converts pathological inflammation to reparative inflammation. This study illustrates that the P2X7R/NLRP3 inflammasome axis is a promising regenerative therapeutic target for enthesis injury treatment.

Regeneration is precisely modulated by dynamic inflammation and cross-talk of cellular components in niches (*11*). Poor regeneration of the enthesis has long been a challenging issue in sports and regenerative medicine and is closely related to the pathological proinflammation and disharmonious cross-talk in niche (*29*, *30*). However, the underlying mechanisms by which the inflammation and cross-talk in niche modulate enthesis regeneration remain undetermined. This research discovers that NLRP3 inflammasomes in macrophages exacerbate inflammation and inhibit enthesis regeneration via IL-1β inflammatory and DTA metabolic cross-talk between macrophages and stem cells. NLRP3 inflammasome–mediated secretome suppresses the proliferation, differentiation, and migration of stem cells. Furthermore, P2X7R in macrophages induces the activation of NLRP3 inflammasomes and suppresses enthesis regeneration. Therefore, this study sheds light on the crucial role of the P2X7R/NLRP3 inflammasome axis in the enthesis regeneration (Fig. 9).

NLRP3 inflammasomes act as a double-edged sword in the innate immune system. On the one hand, NLRP3 inflammasomes help eliminate pathogenic microbial infections (*31*). On the other hand, their aberrant and chronic activation in some pathological conditions contributes to inflammatory disorders, including diabetic complications, rheumatoid arthritis, atherosclerosis, and Alzheimer’s disease (*32*). Another study showed that macrophage pyroptosis induced by NLRP3 inflammasomes accelerated the senescence of TSPCs, exacerbating the formation of heterotopic ossification (*33*). However, it was found that galvanic current activates the NLRP3 inflammasomes and does not induce inflammasome-mediated pyroptosis, promoting the production of collagen I in tendons, indicating that moderate activation of NLRP3 inflammasomes is beneficial for tendon regeneration (*34*). This study demonstrates that the overactivity of NLRP3 inflammasomes in macrophages contributes to the poor enthesis regeneration.

It has been observed that other members of the NLR family, such as NLRC1, NLRP4, and NLRP6, can also assemble into active inflammasomes in response to various endogenous and exogenous stimuli (*35*). Moreover, the cytoplasmic receptor AIM2 detects double-stranded DNA of both microbial and host origin, subsequently assembling to form the AIM2 inflammasome (*36*). Other types of inflammasomes regulate the pathological progression of numerous diseases and tissue regeneration as well (*37*). Fidler *et al.* (*38*) discovered that AIM2 inflammasome activation exacerbates atherosclerosis during clonal hematopoiesis. Jiang *et al.* (*39*) revealed that the AIM2 inflammasome exacerbates tendinopathy, and pristimerin alleviated the progression of tendinopathy by inhibiting the activation of AIM2 inflammasomes. Here, the scRNA-seq showed that *Nlrp3* and *Aim2* were primarily expressed in macrophages, while the expression levels of *Nlrc4*, *Nlrp1b*, and *Nlrp6* were negligible. Although the relative caspase-1 activity in *Nlrp3*^−/−^ mice was restrained after enthesis injury, its levels remained significantly elevated in *Nlrp3*^−/−^ mice at 3 dpi compared to the sham operation. Hence, it can be inferred that, apart from Nlrp3 inflammasomes, other inflammasomes, such as AIM2, may also become activated and regulate enthesis regeneration.

Inflammatory factors are crucial regulators in niche, controlling local inflammation, stem cell behaviors, and the quality of regeneration (*11*). As one of the main end products of NLRP3 inflammasomes, the dominant effect of IL-1β undoubtedly modulates the niche. By inhibiting the β-catenin pathway via IL-1R1/Myeloid Differentiation primary response 88 (MyD88) signaling, IL-1β impaired the capabilities of osteogenic differentiation, migration, and proliferation of stem cells (*21*). IL-1β signaling has also been found to reduce the sensitivity of stem cells to growth factors such as bone morphogenetic protein-2 (BMP-2) and platelet-derived growth factor–BB (PDGF-BB) and accelerate cellular senescence (*22*). Conversely, antagonizing IL-1β signaling restored the sensitivity of stem cells to these growth factors, thereby accelerating bone regeneration (*22*). It was also reported that IL-1β accelerated TSPCs senescence and reduced tenogenic differentiation (*33*). Here, scRNA-seq indicated that IL-1β signaling mediated cross-talk between macrophages and mesenchymal stem cells in the injured enthesis niche. Furthermore, blocking IL-1β signaling with neutralizing antibodies accelerated enthesis regeneration. Therefore, it is concluded that IL-1β acts as an inhibitory inflammation signaling and contributes to poor regeneration of enthesis. Unexpectedly, we discovered that NLRP3 inflammasomes influenced the production of IL-10 and IL-13 in macrophages and injured enthesis. IL-10 and IL-13 are pivotal anti-inflammatory factors that facilitate the polarization of macrophages toward the anti-inflammatory M2 type and inflammatory resolution (*13*, *40*). scRNA-seq and immunofluorescence staining showed that the proportion of AIM was higher in *Nlrp3*^−/−^ mice than in wild-type controls. IL-10 also acts as a regenerative factor that promotes tissue repair and regeneration. Previous studies have demonstrated that IL-10 promotes osteogenic differentiation of mesenchymal stem cells, expedites bone regeneration, and exerts a beneficial impact on enthesis regeneration (*41*, *42*). Therefore, NLRP3 inflammasomes modulate tissue regeneration through diverse inflammatory cytokines.

Moreover, NLRP3 inflammasomes are able to modulate the production of metabolites to regulate stem cell behavior, thereby manipulate tissue regeneration and repair (*23*). Mehrotra *et al.* (*23*) reveal that NLRP3 inflammasome activation induces de novo synthesis of PGE2 and its release through GSDMD pores, which accelerates migration of fibroblasts and wound healing. Chi *et al.* (*43*) identified 11,12-epoxyeicosatrienoic acid (11,12-EET) as a bioactive, prohealing oxylipin secreted from macrophages in a GSDMD-dependent manner. 11,12-EET boosts the activation and proliferation of muscle stem cells through amplifying fibroblast growth factor signaling (*43*). This study illustrates that NLRP3 inflammasomes suppress the production of DTA in injured enthesis and hyperactive BMDMs. In addition, our results indicate that DTA activates phosphatidylinositol signaling to promote cell proliferation and replenishing DTA boosts enthesis regeneration (*44*). Chen *et al.* (*45*) found that DTA elicited antioxidant and anti-inflammatory effects. DTA may have similar healthy beneficial properties like docosahexaenoic acid as they share similar chemical structure (*46*). Considering that NLRP3 inflammasomes induce simultaneous release of inflammatory cytokines and metabolites, the CM of BMDMs was collected to investigate their synergetic effects on stem cells. It was revealed that CM of wild-type BMDMs suppresses the differentiation and migration of stem cells, while the inhibitory effect of the CM of *Nlrp3^−/−^* BMDMs was notably attenuated, indicating that the general effects of NLRP3 inflammasome–mediated secretome are detrimental for stem cells.

For the activation of NLRP3 inflammasomes, priming and activation signals are indispensable (*35*). NLRP3 and IL-1β barely express during physiological conditions, and the critical role of priming signals is to provide substrates for the subsequent activation of inflammasomes (*35*). When cells detect external stimuli via the damage-associated molecular patterns (DAMP)–sensing receptors, the NF-κB pathway is activated, which increases the production of NLRP3 and pro–IL-1β (*35*). Previous studies have illustrated that the priming signal of the NF-κB pathway was involved in the pathogenesis and regeneration of enthesis after injury (*24*). Upon receiving activation signals, NLRP3 inflammasomes assemble and become active (*35*). Now identified activation signals include potassium ion efflux mediated by P2X7R, reactive oxygen species (ROS), monosodium urate, and Oxidized mitochondrial DNA (Ox-mtDNA) (*36*, *47*). This study found that P2X7R was expressed on macrophages in injured enthesis. Conditional KO of *P2rx7* in myeloid cells substantially decreased the activity of NLRP3 inflammasomes. Thus, it is believed that P2X7R induces the activation of NLRP3 inflammasomes following enthesis injury. Previous literature has indicated that mitochondrial dysfunction occurs after tendon and enthesis injuries (*48*, *49*). Mitochondrial dysfunction decreases the cellular capacity for antioxidant stress response, thereby increasing the production of ROS and Ox-mtDNA (*47*). Accordingly, it is speculated that mitochondrial dysfunction following enthesis injury promotes the activation of NLRP3 inflammasomes as well.

Targeting NLRP3 inflammasomes and IL-1β signaling has shown substantial therapeutic potential against numerous inflammatory diseases (*20*). This research reveals that NLRP3 inflammasomes and IL-1β signaling are important contributors to poor enthesis healing and are potential targets for enthesis injury treatment. However, whether inhibiting NLRP3 inflammasomes or blocking IL-1β signaling is more effective in facilitating tissue regeneration remains to be further investigated and discussed. Although IL-1β release mediated by NLRP3 inflammasomes blunts the regeneration, NLRP3 inflammasomes and GSDMD also induced the synthesis and release of prohealing metabolites including PGE2 and 11,12-EET (*23*, *43*). Compared to inhibiting NLRP3 inflammasomes, blocking IL-1β signaling may not only alleviate the dominant proinflammatory effect of NLRP3 inflammasomes but also preserve beneficial metabolites produced by NLRP3 inflammasomes. However, our results indicated that inhibit NLRP3 inflammasomes promote the production of prohealing metabolites as well. Therefore, determining whether targeting NLRP3 inflammasomes or IL-1β signaling is more effective for treating enthesis injuries requires further investigation through large animal studies and clinical trials.

Unexpectedly, this study discovered that global KO of *P2rx7* alters enthesis development, resulting in disorganized fibrocartilage arrangement and reduced bone mass. P2X7R is an ATP-gated plasma membrane ion channel that is expressed in various cell types, including osteoblasts and osteoclasts (*50*). Global KO of P2rx7 significantly reduced bone mass in mice, while activating P2X7R alleviated osteoporosis, indicating that P2X7R participates in bone development and homeostasis (*51*, *52*). Mechanical stimulation promotes ATP release, which subsequently activates P2X7R, thereby enhancing bone homeostasis and increasing bone mass (*53*). Mechanical stimulation is essential for enthesis development, while unloading induces structural changes in the enthesis (*54*, *55*). Therefore, it is speculated that P2X7R may sense mechanical stimulus and influence enthesis development. However, conditional KO of P2rx7 in myeloid cells does not alter enthesis structure, suggesting that P2X7R on myeloid cells does not contribute to enthesis development. Enthesis development is probably regulated by P2X7R on other cells, such as enthesoblasts or tenocytes.

This study has several limitations. First, this study used an acute model of RCTR; however, clinically, the rotator cuff enthesis frequently undergoes chronic degeneration and injury initially before a tear occurs. Consequently, this model does not fully replicate the pathophysiological processes of enthesis injury and regeneration observed in patients. Second, the conclusions of this investigation are based on results from a mouse model, but the regenerative capability of mice is much stronger than that of larger animals or humans. Therefore, the conclusions require further validation in larger animals or humans. Third, given that global KO may alter mouse physiology, using tamoxifen-induced conditional KO of *Nlrp3* in macrophages is preferred for global KO mice. Fourth, considering inflammation is dynamic within the niche, only one time point of scRNA-seq is insufficient to uncover the panorama of inflammation during the whole process of enthesis regeneration. Fifth, IL-1β neutralizing antibodies were used to investigate the impact of IL-1β signaling on enthesis regeneration; however, these antibodies cannot completely block the IL-1β signaling pathway. Thus, it would be better to gain a more precise comprehension of the function of IL-1β signaling in enthesis regeneration with *Il1r1* KO mice. Last, this study did not uncover the specific mechanism of how the NLRP3 inflammasomes regulated the biosynthesis of DTA.

In conclusion, this research illustrates that the P2X7R/NLRP3 inflammasome axis suppresses enthesis regeneration through inflammatory and metabolic cross-talk between macrophages and stem cells. This study provides a previously unidentified perspective on how NLRP3 inflammasomes and inflammation influence enthesis regeneration and highlights that the P2X7R/NLRP3 inflammasome axis and its derived inflammatory cytokines and metabolites are promising niche-directed regenerative therapeutic targets for heterogeneous interface injuries, especially enthesis.

## MATERIALS AND METHODS

### Study design

This study aimed to understand the mechanism of how the P2X7R/NLRP3 inflammasome axis influences enthesis regeneration. To evaluate enthesis regeneration, histological, radiological, and biomechanical tests were carried out. The sample size reflects the number of independent replicates and is presented in the figures. All animal experiments were approved by the ethics review boards.

### Animals and models

The animal experimentation was approved and supervised by the Animal Care and Use Committee of Shanghai Sixth People’s Hospital (Animal Experiment Registration number: DWSY2021-0113 and DWSY2023-0003). Wild-type C57BL/6 mice were purchased from the Shanghai Laboratory Animal Center. *Nlrp3* KO (*Nlrp3^−/−^*) mice (stock no. S-KO-05210) and *P2rx7* KO (*P2rx7 KO*) mice (stock no. S-KO-03541) were obtained from Cyagen (Suzhou, China). *P2rx7-flox* (*P2rx7^f/f^*) mice (strain no. T009615) were purchased from GemPharmatech (Nanjing, China). *Lyz2-Cre* mice (strain no. 004781) were purchased from the Jackson Laboratory (Maine, USA). *Lyz2-Cre:*: *P2rx7^f/f^* mice were generated by crossing *Lyz2-Cre* mice with *P2rx7^f/f^* mice. Primers used for genotyping the *Nlrp3^−/−^*, *P2rx7 KO*, *P2rx7^f/f^*, and *Lyz2-Cre* mice are listed in table S1.

The mouse RCTR model was generated as previously reported (*56*). Briefly, mice aged 12 to 14 weeks were anesthetized with 1% pentobarbital sodium. A 2-cm incision was made to expose the deltoid muscle, and proximal deltoid was peeled off to expose the supraspinatus tendon (fig. S20, A and B). The supraspinatus tendon was elevated with a tissue probe (fig. S20C). Suture attachment was generated with a 6-0 Prolene suture with 9.0 taper point needles in a modified Kessler suture pattern (fig. S20D). The supraspinatus tendon was then transected with a #11 blade close to the great tuberosity (fig. S20E). The remnant enthesis was debrided with a scalpel (fig. S18F). Subsequently, two crossing bone tunnels were created using a 27-gauge needle (fig. S20G). The 6-0 Prolene suture was then passed through each tunnel and tied in a knot to repair the rotator cuff (fig. S20H). Mice with an incision that was only made on the skin of shoulder were the sham surgery group. Surgeries were operated after at least 1 week of accommodation to the local environment. Mice were freely mobile within cages and had continuous access to food and water.

For antibodies treatment experiments, 200 μg of IL-1β neutralizing antibodies (BioXcell, New Hampshire, USA, BE0246) or control IgG (BioXcell, New Hampshire, USA, BE0091) was injected into the joint cavity near the injured enthesis at 3 and 7 dpi. IL-1β neutralizing antibodies were labeled with fluorescent secondary antibodies (Abcam, ab173004) and visualized with an in vivo imaging system (IVIS) (PerkinElmer, IVIS spectrum). As for the metabolite replenished experiment, 1 μg of docosatrienoic acid (MedChemexpress, New Jersey, USA, HY-101408) or saline was injected into the surrounding area of the injured enthesis at 3 and 7 dpi. The mice were humanely euthanized at 14 and 28 dpi.

### Histological, IHC, and immunofluorescent staining

Mice were euthanized, and rotator cuffs were harvested at 3, 7, 14, and 28 dpi. Tissues were fixed with 4% paraformaldehyde for 24 hours and then decalcified in 10% EDTA for 14 days. The tissues were then dehydrated and embedded in paraffin. Four-micrometer-thick sections were obtained in the coronal plane through the greater tuberosity. H&E and toluidine blue staining were performed through conventional protocol. The regeneration of the enthesis was evaluated by two blinded observers according to the modified tendon maturing score as previously reported (*56*). The details of the modified tendon maturing score are listed in table S2. The higher scores indicated better enthesis regeneration.

For IHC staining, the sliced sections were rehydrated in alcohol gradients and subjected to antigen retrieval using sodium citrate. The sections were incubated with 3% hydrogen peroxide, blocked with 10% serum, and incubated with antibodies overnight. Then, the sections were incubated with biotinylated secondary antibodies, and chromogenic substrate was added at the end. Histological and IHC sections were observed in a Leica DM4000B microscope (Germany). The metachromasia area (fibrocartilage) of toluidine blue staining and the sum IOD of IHC staining were quantified by ImageJ software (National Institutes of Health, USA).

For immunofluorescence staining, we used a four-color multiple immunofluorescence kit (Recordbio Biological Technology, Shanghai, China) based on the tyramide signal amplification technology according to the manufacturer’s protocol. The immunofluorescence staining sections were scanned by Zeiss LSM980 (Germany). The numbers of positive cells in each group were counted in the region of interest (ROI) and normalized to the number per square millimeter. All the primary antibodies used in this research are listed in table S3.

### RNA sequencing

Wild-type mice undergoing supraspinatus RCTR at 3 dpi were the experimental group, while mice that experienced sham surgery were the control group. To obtain enthesis tissue, we first isolate the supraspinatus–humeral head complex (fig. S21A). Then, we carefully separate the cortical bone of the greater tuberosity of the humeral head using microsurgical instruments (fig. S21B). After that, we transect the distal one-third of the supraspinatus tendon and isolate the enthesis tissue (fig. S21C). Total RNA from the enthesis was extracted using TRIzol (Invitrogen, CA). mRNA was separated from total RNA using oligo (dT) magnetic beads. Fragmentation buffer was used for splitting mRNA into small fragments of approximately 300 base pairs, which were then separated by magnetic beads. The small mRNA fragments were then used as templates for reverse transcriptase–catalyzed cDNA synthesis. After the addition of adapters to the cDNA, a cDNA library was created through PCR. The Illumina sequencing platform (Illumina NovaSeq 6000, Majorbio, Shanghai, China) was used for pair-end sequencing. PCA, volcano plots, GO enrichment analysis, and heatmaps of gene expression were plotted using Origin 2021. The expression of differential genes was validated by RT-qPCR, IHC staining, and immunofluorescence staining in mice.

### Single-cell RNA sequencing

Enthesis at 7 dpi from wild-type and *Nlrp3^−/−^* mice were carefully harvested under surgical microscopes and pooled together. scRNA-seq was performed by Shanghai Xu Ran Bio Biotechnology Co. Ltd. according to the Chromium Next GEM Single Cell 3′ Kit v3 (10x Genomics, PN-1000215) manufacturer’s recommendations. The cell survival rate is typically above 80% for quality control and enumeration of single cell suspension. After the cells passed quality control, they were rinsed and resuspended to prepare a suitable cell concentration of 700 to 1200 cells/μl for 10x Genomics Chromium. GEMs (gel beads in emulsion) were constructed for single-cell separation and collected for reverse transcription in a PCR machine for labeling. The amplified cDNA was purified by magnetic beads, and then subjected to cDNA amplification. The Gene Expression Library was constructed with the quality-qualified cDNA. The final library pool was sequenced on the Illumina Nova 6000 instrument using 150-bp paired-end reads.

### Micro-CT analysis

After fixation, the whole shoulder was imaged by the micro-CT system (SkyScan 1176, Bruker, Germany). The scanning accuracy was 9 μm, and the scanning voltage was 90 kV. The ROI of the enthesis was selected by a rectangle with a fixed dimension, and the images were analyzed by DataViewer (fig. S22, A to D). The ROI used for bone morphometry analysis is selected from the top to the bottom of the transverse images. BV/TV, BMD, Tb.N, BS/BV, Tb. Th, and Tb.Sp were calculated by CTAn software.

### Biomechanical tests

Supraspinatus tendon-humerus complexes were used for biomechanical tests. The tendon was glued between two pieces of sandpaper, and the humerus was fixed in a centrifugal tube (2 ml) (fig. S23A). The tendon and the humerus were immobilized with clamps, respectively (fig. S23B). The biomechanical properties of tendon-humerus complexes were tested by the Mcro Biomechanical Testing System (XHD-HSS100, China). The rupture of the tendon-humerus complexes indicated the completion of the test (fig. S23C). Failure load, work, and stiffness were calculated through load-deformation curves. The cross-sectional area of the enthesis was determined through micro-CT images. Samples were preloaded to 0.01 N, and gauge length (*L*_0_) was measured as the distance between the enthesis and the edge of sandpaper. Strain was calculated as the deformation divided by the *L*_0._ Maximum stress, Young’s modulus, and toughness were calculated through stress-strain curves.

### Primary culture of BMDMs

C57BL/6 mice aged 8 weeks were euthanized to isolate BMDMs. Briefly, the bone marrow of femurs and tibiae was flushed out by minimum essential medium α (α-MEM), and erythrocytes were removed by erythrocytes lysis buffer (Yeasen, China). Then, the bone marrow was cultured with H-DMEM (high-glucose Dulbecco’s modified Eagle’s medium) complete medium containing macrophage colony-stimulating factor (40 ng/ml; Novoprotein, China) for 7 days. Adherent cells were considered BMDMs and used for the subsequent experiment.

For NLRP3 inflammasome activation, BMDMs were firstly treated with LPS (200 ng/ml; Yeasen, China) for 12 hours and then treated with 3 mM ATP (Yeasen, China) for 2 hours. The supernatant was used for subsequent ELISA, mouse inflammation array Q1, untargeted metabolomics, and CM. The total RNA was obtained for RNA sequencing.

### EIF collection

The enthesis was isolated and placed on a 40-μm strainer fixed in a 50-ml tube. Samples were centrifuged at 50*g* for 5 min at 4°C to clear the surface liquid and then at 600*g* for 10 min at 4°C to collect the interstitial fluid for untargeted metabolomics.

### Untargeted metabolomics

#### Sample extraction

The sample stored at an −80°C refrigerator was thawed on ice. The thawed sample was homogenized by a grinder (30 Hz) for 20 s. A 400-μl solution (methanol: water = 7:3, v/v) containing the internal standard was added in to the 100-μl supernatant or EIF, followed by spinning the mixture for 5 min and subsequently centrifugating at 12,000 rpm for 10 min (4°C). The 300-μl supernatant was collected and placed in −20°C for 30 min. The sample was then centrifuged at 12,000 rpm for 3 min (4°C). Two hundred–microliter aliquots of the supernatant were used for liquid chromatography–mass spectrometry (LC-MS) analysis.

#### HPLC conditions

We used two LC-MS methods for all samples. One aliquot was analyzed through positive ion conditions and was eluted from the T3 column (Waters ACQUITY Premier HSS T3 Column 1.8 μm, 2.1 mm by 100 mm) with solvent A (0.1% formic acid in water) and solvent B (0.1% formic acid in acetonitrile) in the following gradient: 5 to 20% in 2 min, increased to 60% in the following 3 min, increased to 99% in 1 min, and remained for 1.5 min, then came back to 5% mobile phase B within 0.1 min, and held for 2.4 min. The analytical conditions were as follows: column temperature, 40°C; flow rate, 0.4 ml/min; injection volume, 4 μl. Another aliquot was analyzed with negative ion conditions and was the same as the elution gradient of the positive mode.

### Mass spectrometer settings

A high-resolution tandem mass spectrometer, Q-Exactive (Thermo Fisher Scientific, Vanquish), was used to detect metabolites eluted from the column. All the methods alternated between full-scan MS and data-dependent Tandem MS (MSn) scans using dynamic exclusion. MS analyses were carried out using electrospray ionization in the positive ion mode and negative ion mode with full-scan analysis over mass/charge ratio 75 to 1000 at 35000 resolutions. Additional MS settings were as follows: ion spray voltage, 3.5 or 3.2 kV in positive or negative mode, respectively; sheath gas, 30 arbitrary units (Arb); auxiliary gas, 5 Arb; ion transfer tube temperature, 320°C; vaporizer temperature, 300°C; collision energy, 30, 40, and 50 V; signal intensity threshold, 1 × 10^6^ cps; top N versus top speed, 10; exclusion duration, 3 s.

### Culture of stem cells

Human BMSCs (CP-H166) and mouse tendon-derived stem cells (TDSCs; CP-M176) were purchased from Pricella (Wuhan, China). The cells were cultured in an incubator at 37°C with α-MEM (Gibco, USA) containing 10% fetal bovine serum (FBS; Cyagen, China). The culture medium was refreshed every 2 days. BMSCs were treated with 50 μM docosatrienoic acid for 48 hours, and then total RNA was isolated for RNA-seq.

### Differentiation, migration, and proliferation of stem cells

BMSCs were seeded in six-well culture plates until 80% confluence. The osteogenic differentiation medium (Cyagen, China) then replaced the culture medium for osteogenic induction. Osteogenic differentiation medium with 5% supernatant of BMDMs from wild-type and *Nlrp3^−/−^* mice was wild-type and *Nlrp3^−/−^* CM. ALP staining was performed using the ALP Assay Kit (Beyotime, China) after 7 days of induction, and ARS staining was performed with 2% ARS solution (Beyotime, China) after 14 days of induction. RT-qPCR was conducted after 14 days of induction.

BMSC pellets were used to evaluate the effect of IL-1β and neutralizing antibodies on the chondrogenic differentiation of stem cells, as previously reported (*57*). Pellets were treated with 5% supernatant of BMDMs from wild-type and *Nlrp3^−/−^* mice. Pellets without treatment served as the control group. After 3 weeks of incubation, Alcian blue, Safranin O, and collagen II IHC were used to evaluate the chondrogenic differentiation of BMSC pellets. The volume and Bern score of pellets were calculated as described previously (*57*). The details of the Bern score are listed in table S4. RT-qPCR was used to assess chondrogenic marker gene expression.

Mouse TDSCs were used to evaluate the tenogenic differentiation of stem cells. H-DMEM (Gibco, USA) with vitamin C (50 μg/ml) and 10% FBS served as the tenogenic medium. The control group included TDSCs cultured in a tenogenic medium, while the experimental groups included TDSCs cultured in a tenogenic medium with 5% supernatant of BMDMs from wild-type and *Nlrp3^−/−^* mice. Sirius red stain and RT-qPCR were used to analyze the tenogenic differentiation of stem cells.

The migration of stem cells was assessed through a transwell assay. BMSCs were seeded in the upper chamber of transwell inserts (Corning, USA) with serum-free α-MEM. Cells cultured in complete medium without other additions in the lower chamber were considered the control group, while the experimental group included cells cultured in complete medium with 5% supernatant of BMDMs from wild-type and *Nlrp3^−/−^* mice. The inserts were fixed with paraformaldehyde and stained with crystal violet (Beyotime, China) after 12 hours of incubation. EdU assay was used to assess stem cell proliferation according to the instruction of the BeyoClick EdU Cell Proliferation Kit (C0078S, Beyotime, China). The images were captured by a microscope and analyzed by ImageJ software.

### Enzyme-linked immunosorbent assay

The concentration of IL-1β in the supernatant from the BMDMs was measured with an ELISA kit (A1010A0201, BioTNT, China) according to the instructions. The enthesis samples were obtained immediately after euthanasia. Samples were lysed in radioimmunoprecipitation assay (RIPA) buffer (Beyotime, China) to acquire the total protein. The concentration of the total protein was quantified with a bicinchoninic acid (BCA) protein assay kit (Beyotime, China). The concentrations of IL-1β, IL-10, and IL-13 of enthesis were measured with ELISA kits (IL-1β: A1010A0201, BioTNT, China; IL-10: A1010A0210, BioTNT, China; IL-13: A1010A0213, BioTNT, China;).

### Mouse inflammation array Q1

The supernatant of wild-type and *Nlrp3^−/−^* BMDMs was collected after treatment with LPS and ATP as described above. Mouse inflammation array Q1 was used to measure the concentration of multiple inflammatory cytokines according to the product instructions.

### Caspase-1 activity detection assay

Caspase-1 activity of enthesis samples was detected with a caspase-1 activity assay kit (C1101, Beyotime, China) according to the protocol. Briefly, enthesis samples were lysed with lysis buffer. The protein concentration of the lysate was measured by a Bradford Protein Assay Kit (P0006, Beyotime, China). The lysate was incubated with Ac-YVAD-pNA for 1 hour and measured at 405 nm in the microplate reader (MPRA9600, Thomas Scientific, USA). Caspase-1 activities were calculated through the standard curve. The relative caspase-1 activity at each time point was assessed by comparing it with that of the sham group.

### Real-time quantitative polymerase chain reaction

Total RNA was extracted from cells with the EZ-press RNA Purification Kit (EZBioscience, USA) according to the instructions. RNA was reverse-transcribed to cDNA via a reverse transcription kit (EZBioscience, USA). SYRB Green qPCR Master Mix (EZBioscience, USA) and the Real-Time PCR system (LightCycler 480, Germany) were used to perform qPCRs. After the initial denaturation at 95°C, 40 cycles of denaturation for 15 s at 95°C and extension for 30 s at 60°C followed. The reference gene was glyceraldehyde-3-phosphate dehydrogenase (*GAPDH*). The primers for *GAPDH*, osteocalcin (*OCN*), collagen type 1 alpha 1 (*Col1a1*), Runt-related transcription factor 2 (*Runx2*), aggrecan (*Agg*), collagen type 2 alpha 1 (*Col2a1*), SRY-Box transcription factor 9 (*Sox9*), scleraxis (*Scx*), *Tnmd*, mohawk homeobox (*Mkx*), IL-1β (*Il1b*), *Nlrp3*, caspase-1 (*Caspase-1*), and P2X purinoceptor 7 (*P2rx7*) were purchased from Sangon (China). The primer sequences were listed in table S5. The gene expression fold change was calculated through the comparative ΔCT method.

### Western blot

BMSCs were treated with 50 μM docosatrienoic acid for 48 hours and then lysed in RIPA lysis buffer (Beyotime, China). Total proteins were separated by 12% SDS–polyacrylamide gel electrophoresis (Beyotime, China) and transferred to polyvinylidene difluoride (PVDF) membranes (Beyotime, China). After incubating with the primary antibody, the PVDF membranes were incubated with the horseradish peroxidase–labeled secondary antibodies (Beyotime, China), and signals were captured using a gel imaging system.

### Statistical analysis

The experimental data were analyzed and visualized with Origin 2021 software. Data are presented as means ± SD. The unpaired two-tailed Student’s *t* test was used for two group comparisons. For comparisons of more than two groups, one-way analysis of variance (ANOVA) followed by Tukey’s multiple comparisons tests was performed. Differences were considered significant when *P* < 0.05.
