# Supplementary material for: The P2X7R/NLRP3 inflammasome axis suppresses enthesis regeneration through inflammatory and metabolic macrophage-stem cell cross-talk
Source: Sci Adv. 2025 Apr 25;11(17):eadr4894. doi: 10.1126/sciadv.adr4894 (PMC12024643; doi:10.1126/sciadv.adr4894)
Supplement: Supplementary file 1 — Figs. S1 to S23 Tables S1 to S5 [file sciadv.adr4894_sm.pdf]

Science Advances
AAAS

Supplementary Materials for

The P2X7R/NLRP3 inflammasome axis suppresses enthesis regeneration through inflammatory and metabolic macrophage-stem cell cross-talk

Haihan Gao *et al.*

Corresponding author: Xin Ma, maxin@sjtu.edu.cn; Shen Liu, liushensjtu@126.com;
Jia Jiang, jessicajj19@sjtu.edu.cn

Sci. Adv. **11**, eadr4894 (2025)
DOI: 10.1126/sciadv.adr4894

This PDF file includes:

Figs. S1 to S23
Tables S1 to S5

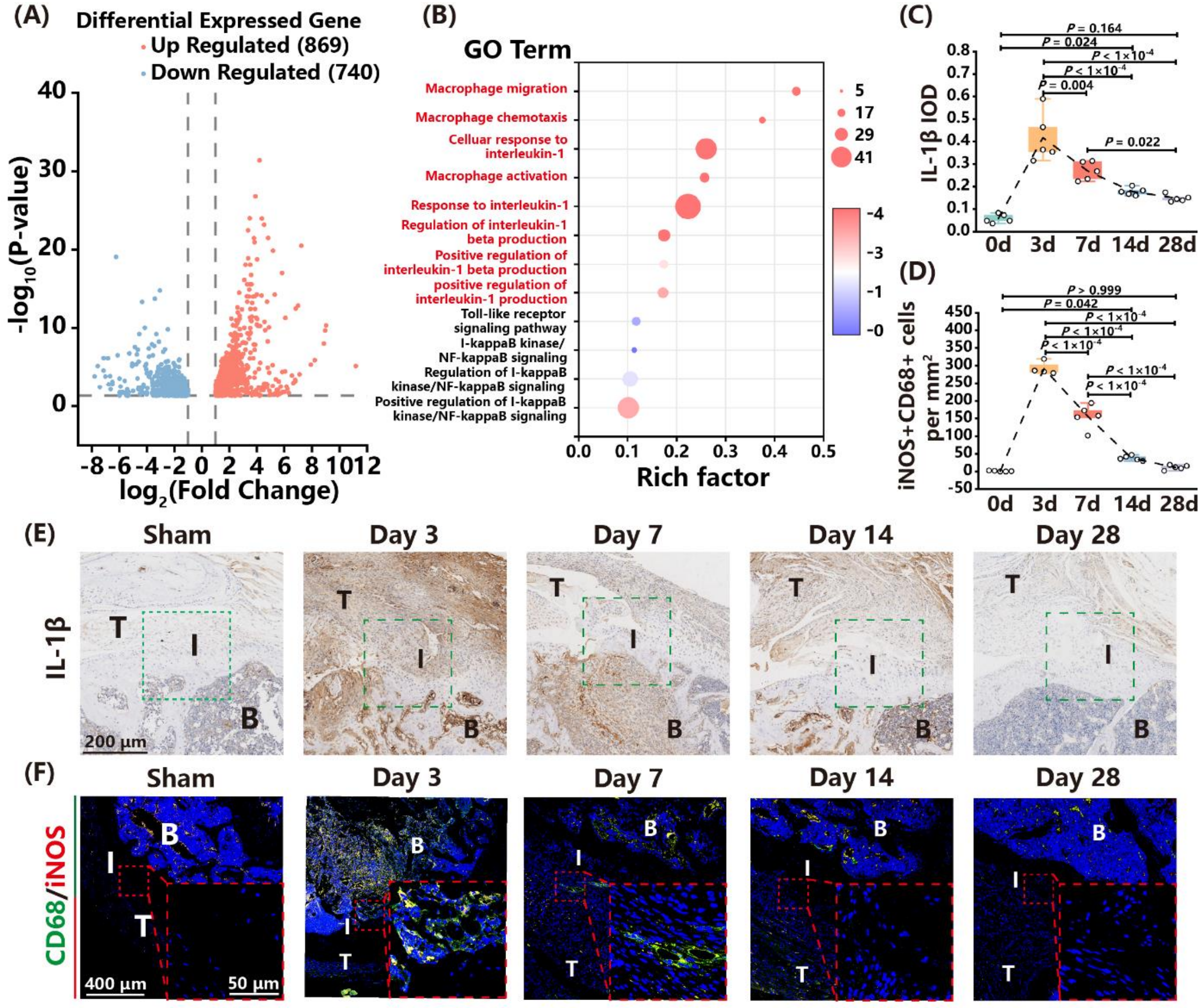

Fig S1. Enthesis injury leads to macrophage infiltration and elevated expression of *Nlrp3, Caspase-1*, and *Il1b* in the mouse model. (A) Volcano plot of differentially expressed genes between the sham operation and RCTR groups. (B) GO enrichment analysis of differentially expressed pathways. Important GO terms are highlighted in red. (C and D) Immunohistochemical staining and IOD of IL-1β in enthesis with sham operation and injured enthesis at 3, 7, 14, and 28 dpi. Green dashed squares represent the enthesis. (E and F) Immunofluorescence staining and quantification of CD68 (green) and iNOS (red) positive cells in enthesis with sham operation and injured enthesis at 3, 7, 14, and 28 dpi. Red dashed squares represent the enlarged images of the enthesis. T: tendon, I: tendon-to-bone interface, B: bone. Data are presented as means ± SD. Statistical significance was determined using one-way analysis of variance (ANOVA) with Tukey's multiple comparisons test and Student's t-test.

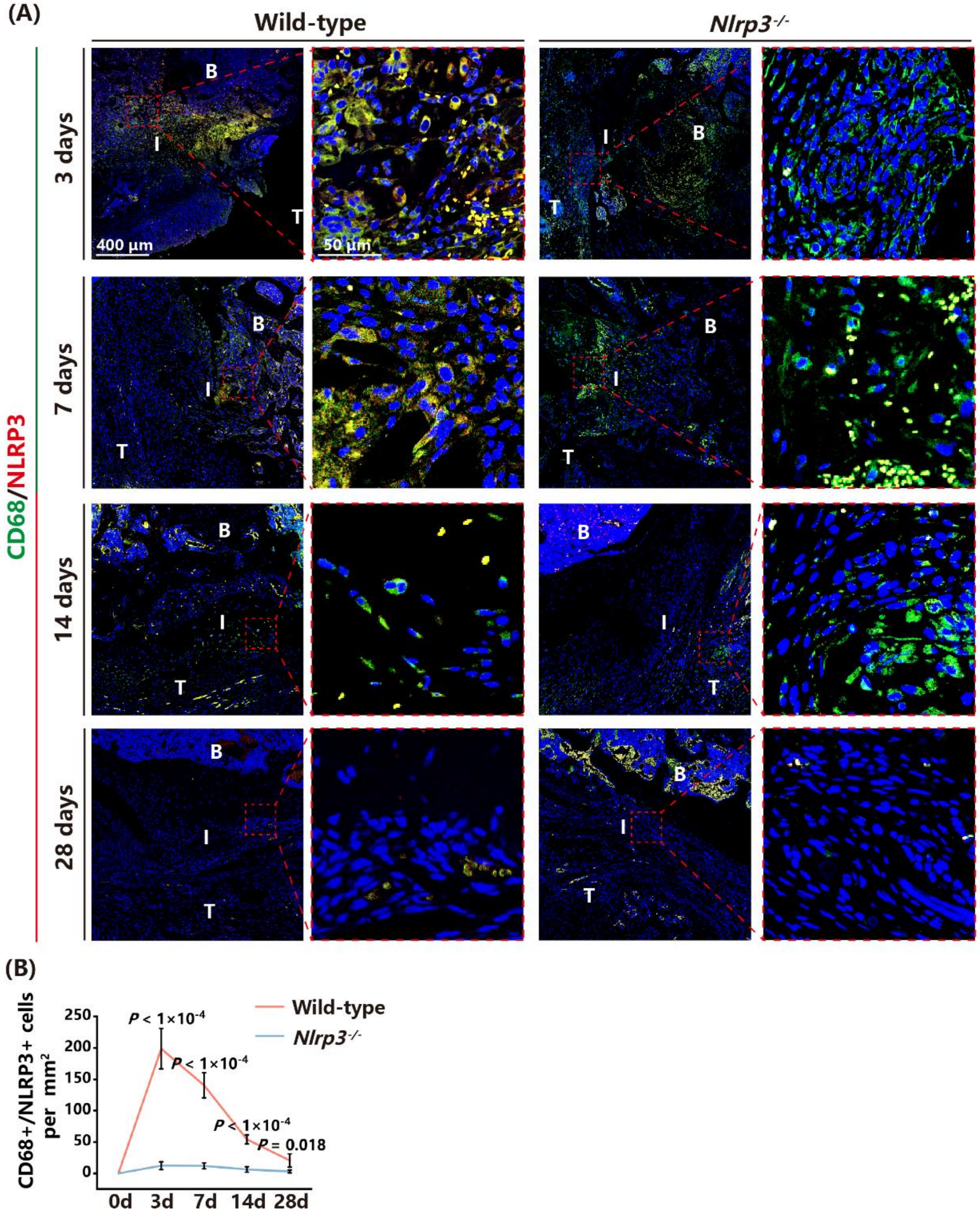

Fig S2. NLRP3 is primarily expressed in macrophages. (A) Immunofluorescence staining of CD68 (green) and NLRP3 (red) positive cells in the enthesis of wild-type and *Nlrp3*$^{-/-}$ mice at 3, 7, 14, and 28 dpi. Red dashed squares represent the enlarged images of the enthesis. (B) Quantification of CD68-positive and NLRP3-positive cells in the enthesis of wild-type and *Nlrp3*$^{-/-}$ mice at 3, 7, 14, and 28 dpi. T: tendon, I: tendon-to-bone interface, B: bone. Data are presented as means ± SD. Statistical significance was determined using one-way analysis of variance (ANOVA) with Tukey's multiple comparisons test.

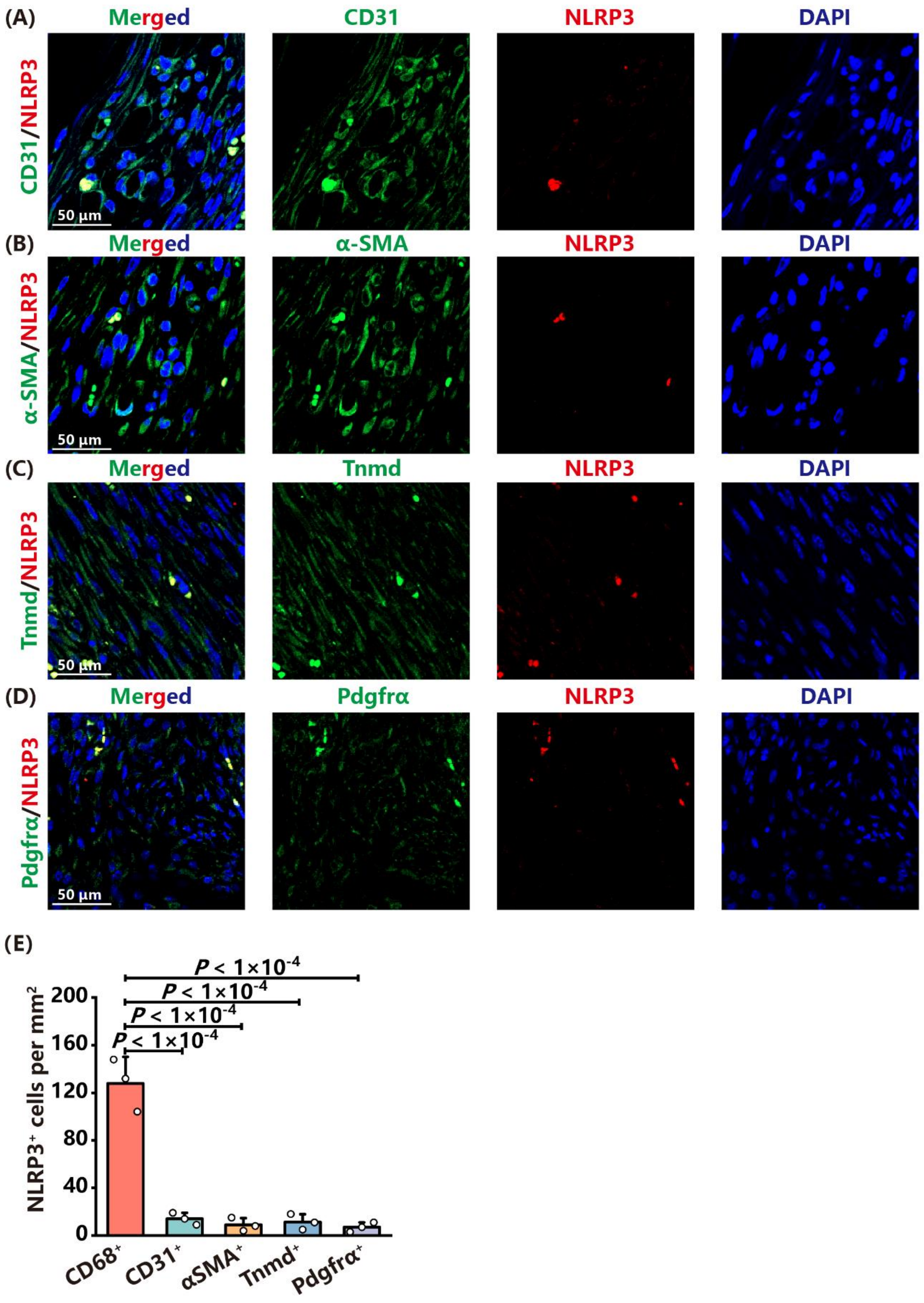

Fig S3. The expression of NLRP3 in various cell types. (A) Immunofluorescence staining of NLRP3 (red) and CD31 (green) in the injured enthesis. (B) Immunofluorescence staining of NLRP3 (red) and α-SMA (green) in the injured enthesis. (C) Immunofluorescence staining of NLRP3 (red)

and Tnmd (green) in the injured enthesis. (D) Immunofluorescence staining of NLRP3 (red) and Pdgfrα (green) in the injured enthesis. (E) Quantification of NLRP3-positive cells in different cell types. Data are presented as means ± SD. Statistical significance was determined using one-way analysis of variance (ANOVA) with Tukey's multiple comparisons test.

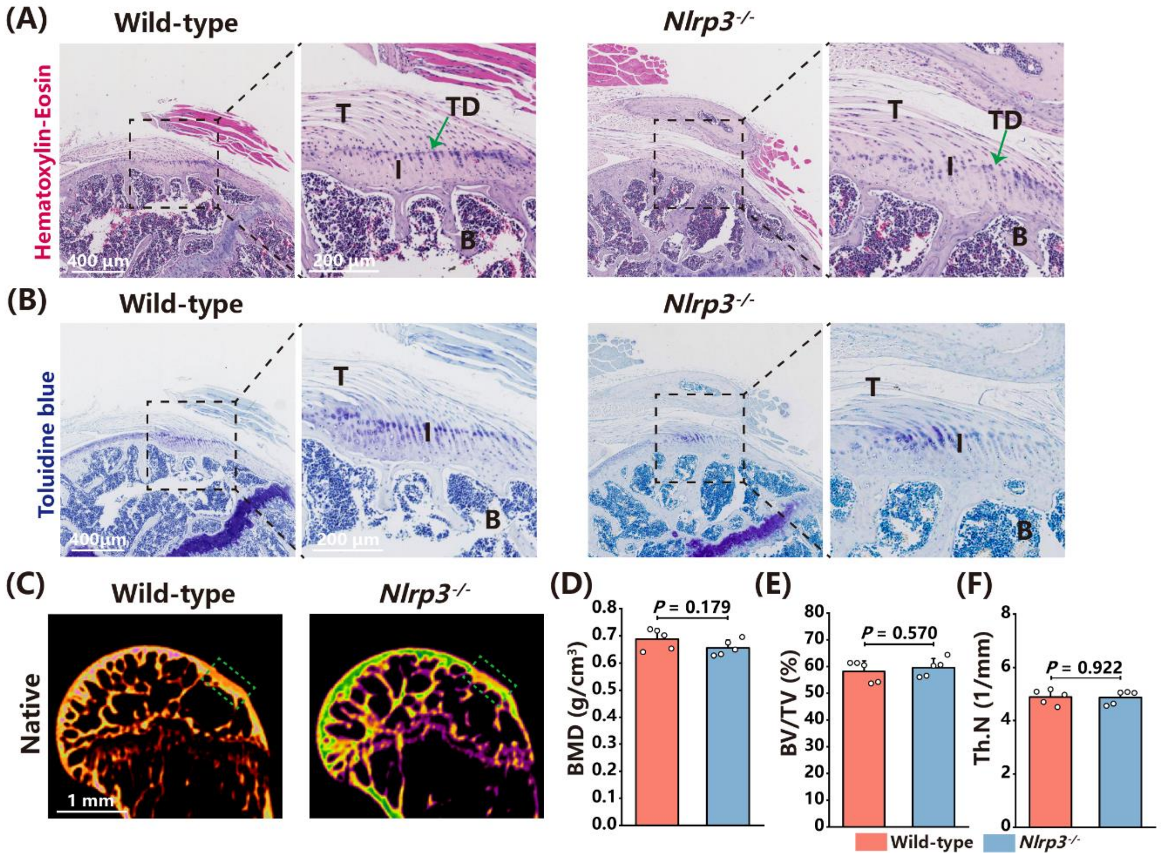

Fig S4. The native enthesis of wild-type controls and *Nlrp3*$^{-/-}$ mice. (A and B) H&E and toluidine blue staining of the enthesis in wild-type and *Nlrp3*$^{-/-}$ mice. Black dashed squares represent the enlarged images of the enthesis. (C) Micro-CT of the native enthesis in wild-type and *Nlrp3*$^{-/-}$ mice. Green dashed squares represent the area of the enthesis. (D-F) Quantitative analysis of the BMD, BV/TV, and Tb. N of native enthesis in wild-type and *Nlrp3*$^{-/-}$ mice. T: tendon, I: tendon-to-bone interface, B: bone, TD: tidemark. Data are presented as means ± SD. Statistical significance was determined using Student's t-test.

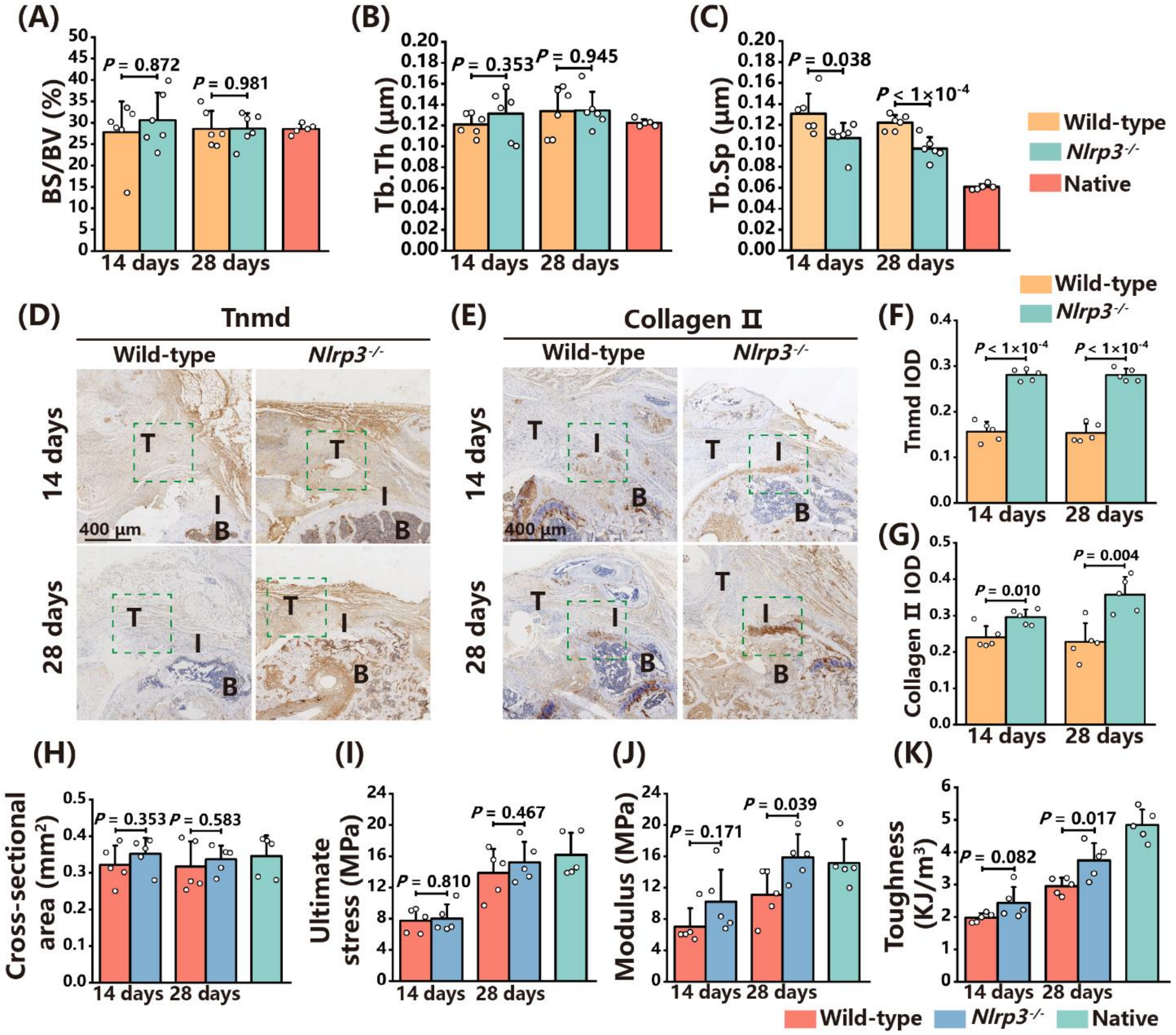

Fig S5. The activation of NLRP3 inflammasomes inhibits enthesis regeneration. (A-C) Quantitative analysis of BS/BV, Tb.Th, and Tb.Sp of the enthesis. (D-G) Immunohistochemical staining and IOD of Tnmd and collagen II in injured enthesis at 14 and 28 dpi. Green dashed squares represent areas used for quantification. (H-K) Cross-sectional area, ultimate stress, modulus, and toughness of the enthesis in wild-type and *Nlrp3*$^{-/-}$ mice at 14 and 28 dpi. T: tendon, I: tendon-to-bone interface, B: bone. Data are presented as means ± SD. Statistical significance was determined using one-way analysis of variance (ANOVA) with Tukey's multiple comparisons test and Student's t-test.

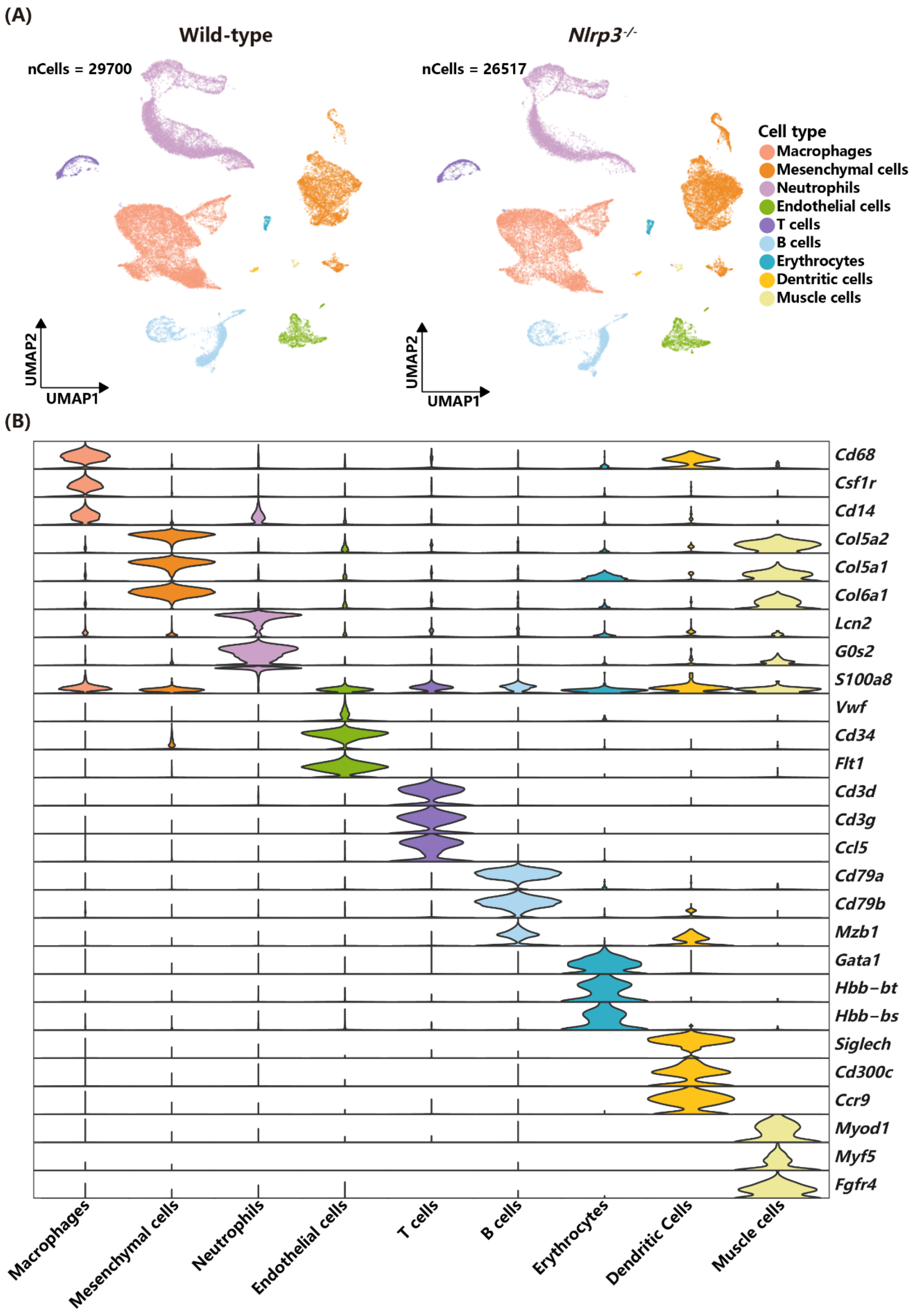

Fig S6. scRNA-seq of the injured enthesis from wild-type controls and *Nlrp3*$^{-/-}$ mice. (A) UMAP plots of 29,700 cells from wild-type controls (n = 3) and 26,517 cells from *Nlrp3*$^{-/-}$ mice (n = 3). (B) Violin plots of marker genes expression.

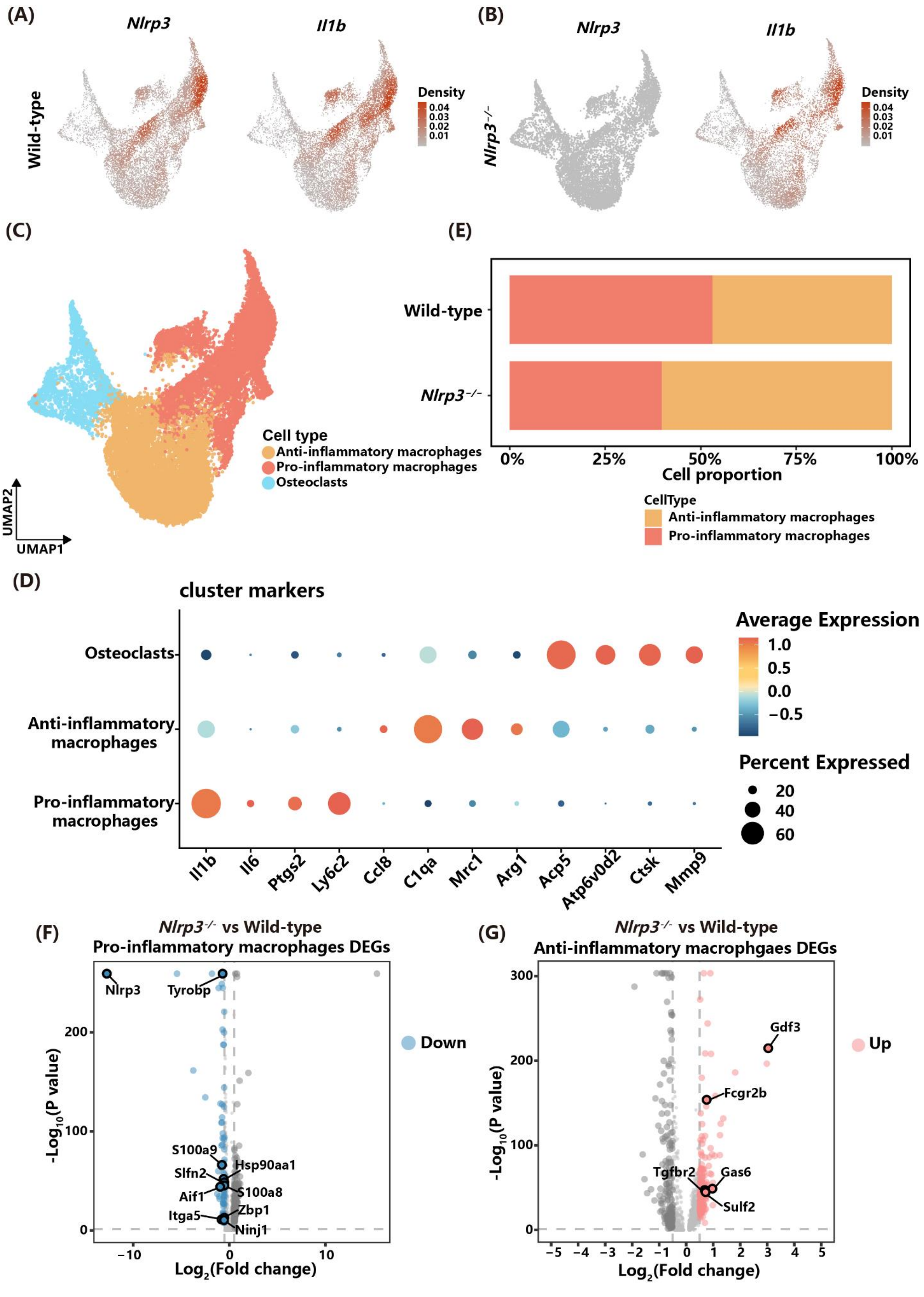

(A)
Nlrp3
Il1b
Wild-type
Density
0.04
0.03
0.02
0.01
(B)
Nlrp3
Il1b
Nlrp3−/−
Density
0.04
0.03
0.02
0.01
(C)
Cell type
Anti-inflammatory macrophages
Pro-inflammatory macrophages
Osteoclasts
UMAP2
UMAP1
(E)
Wild-type
Nlrp3−/−
0%
25%
50%
75%
100%
Cell proportion
CellType
Anti-inflammatory macrophages
Pro-inflammatory macrophages
(D)
cluster markers
Osteoclasts
Anti-inflammatory macrophages
Pro-inflammatory macrophages
Il1b
Il6
Ptgs2
Ly6c2
Ccl8
C1qa
Mrc1
Arg1
Acp5
Atp6v0d2
Ctsk
Mmp9
Average Expression
1.0
0.5
0.0
−0.5
Percent Expressed
20
40
60
(F)
Nlrp3−/− vs Wild-type
Pro-inflammatory macrophages DEGs
Nlrp3
Tyrobp
S100a9
Hsp90aa1
Slfn2
S100a8
Aif1
Zbp1
Itga5
Ninj1
Down
-Log10(P value)
Log2(Fold change)
(G)
Nlrp3−/− vs Wild-type
Anti-inflammatory macrophgaes DEGs
Gdf3
Fcgr2b
Tgfbr2
Gas6
Sulf2
Up
-Log10(P value)
Log2(Fold change)

Fig S7. Characterization of macrophages in wild-type controls and *Nlrp3*$^{-/-}$ mice. (A) Feature plots displaying the single cell gene expression of *Nlrp3* and *Il1b* in wild-type and *Nlrp3*$^{-/-}$ mice. (C) UMAP plot of macrophages from wild-type controls and *Nlrp3*$^{-/-}$ mice. (D) Dot plot of marker genes expression. Dot hue represents the average gene expression. Dot size represents the fraction of a cell subset expressing a given gene. (E) Proportions of pro-inflammatory macrophages and anti-inflammatory macrophages in wild-type controls and *Nlrp3*$^{-/-}$ mice. (F) Volcano plot of DEGs in pro-inflammatory macrophages. Blue dots indicate the down-regulated genes. (G) Volcano plot of DEGs in anti-inflammatory macrophages. Red dots indicate the up-regulated genes.

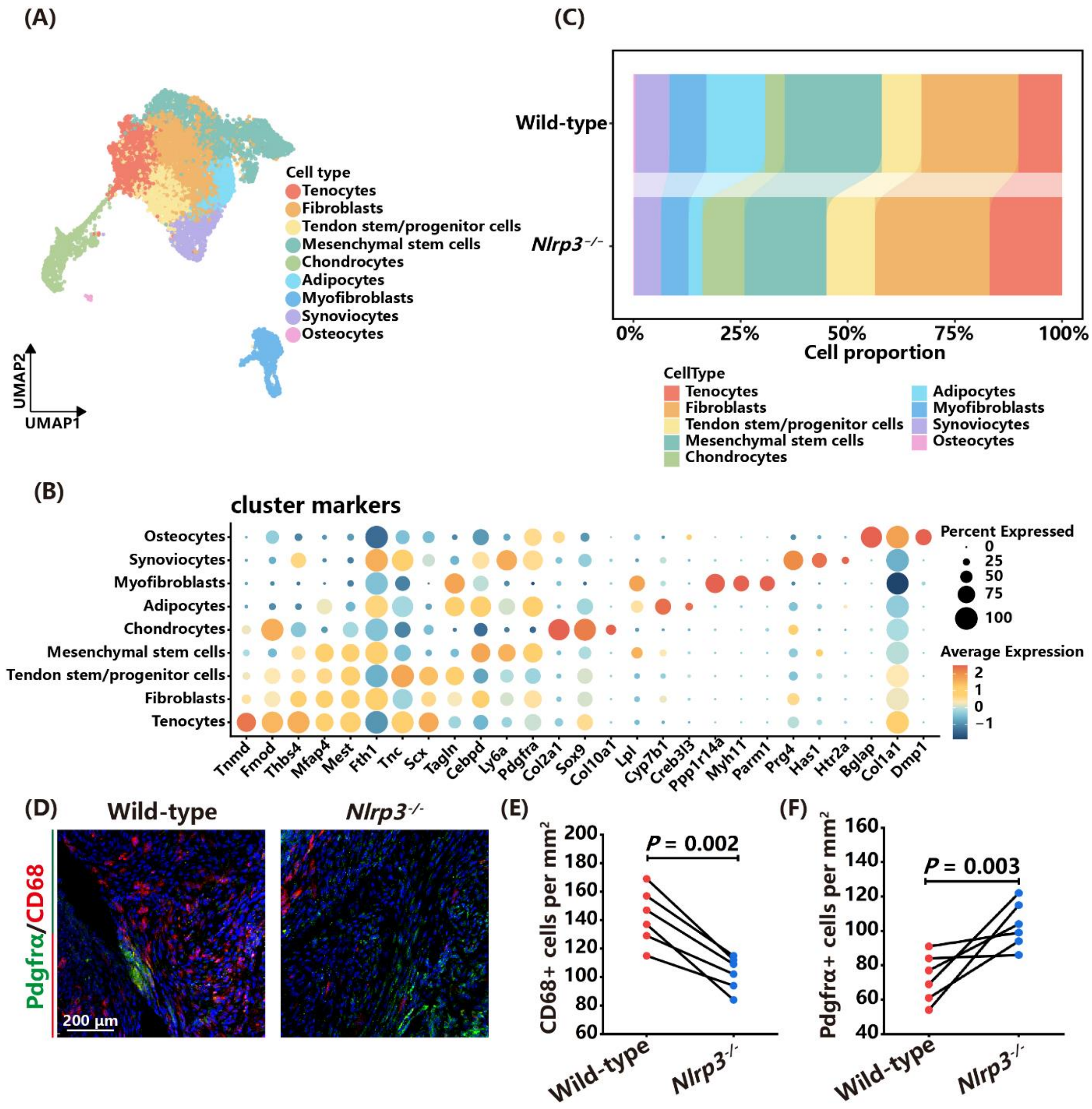

Fig S8. Characterization of mesenchymal cells in wild-type controls and *Nlrp3*$^{-/-}$ mice. (A) UMAP plot of mesenchymal cells from wild-type controls and *Nlrp3*$^{-/-}$ mice. (B) Dot plot of marker genes expression. Dot hue represents the average gene expression. Dot size represents the fraction of a cell subset expressing a given gene. (C) Proportion of tenocytes, fibroblasts, tendon stem/progenitor cells, mesenchymal stem cells, chondrocytes, adipocytes, myofibroblasts, synovial cells, and osteocytes in wild-type controls and *Nlrp3*$^{-/-}$ mice. (D-F) Immunofluorescence staining and quantification of CD68 (red) and Pdgfrα (green) positive cells in the enthesis of wild-type controls and *Nlrp3*$^{-/-}$ mice at 7 dpi. Statistical significance was determined using Student's t-test.

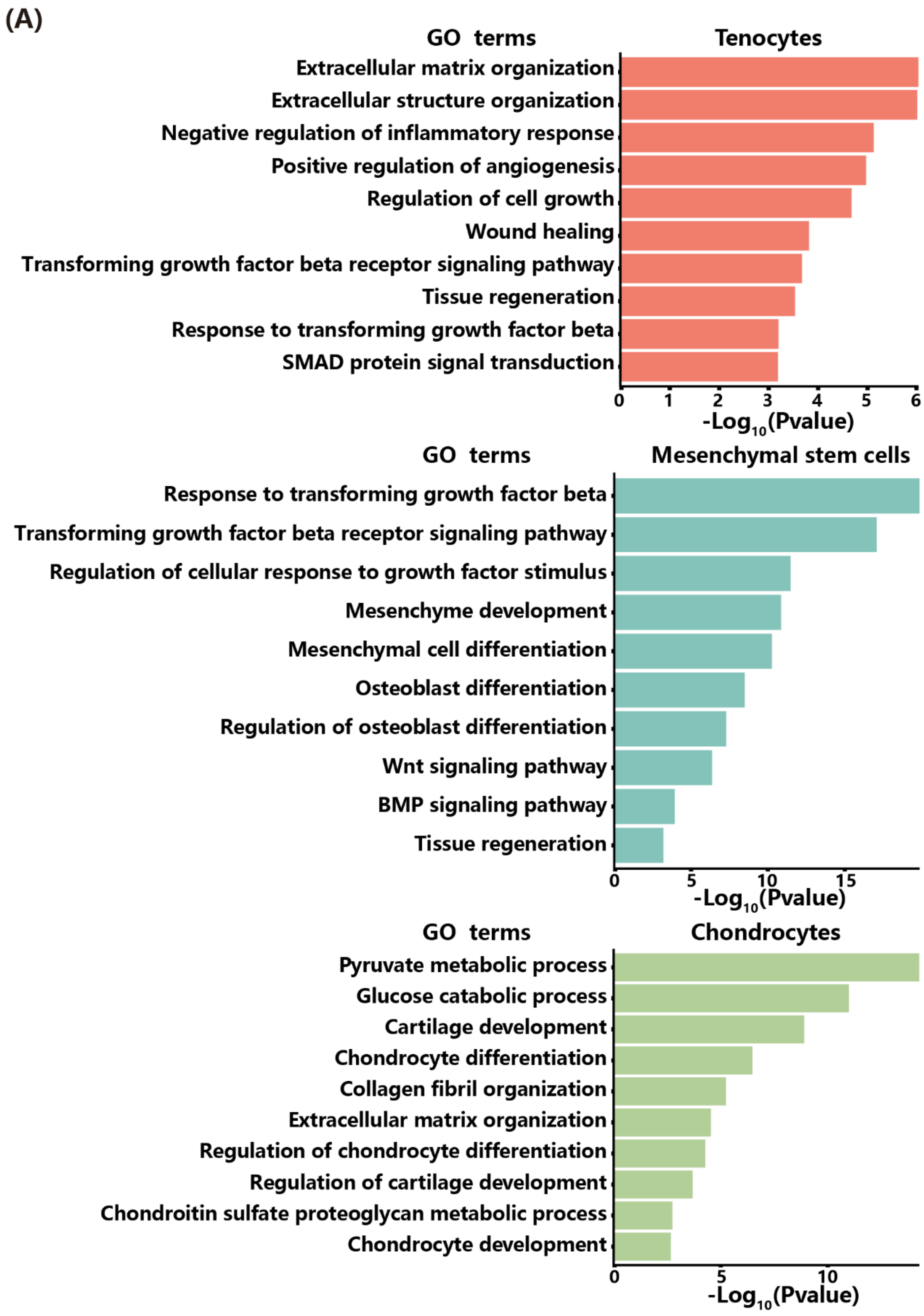

Fig S9. GO enrichment of mesenchymal cells subsets. (A) GO enrichment of up-regulated genes in tenocytes, mesenchymal stem cells, and chondrocytes of *Nlrp3*$^{-/-}$ mice.

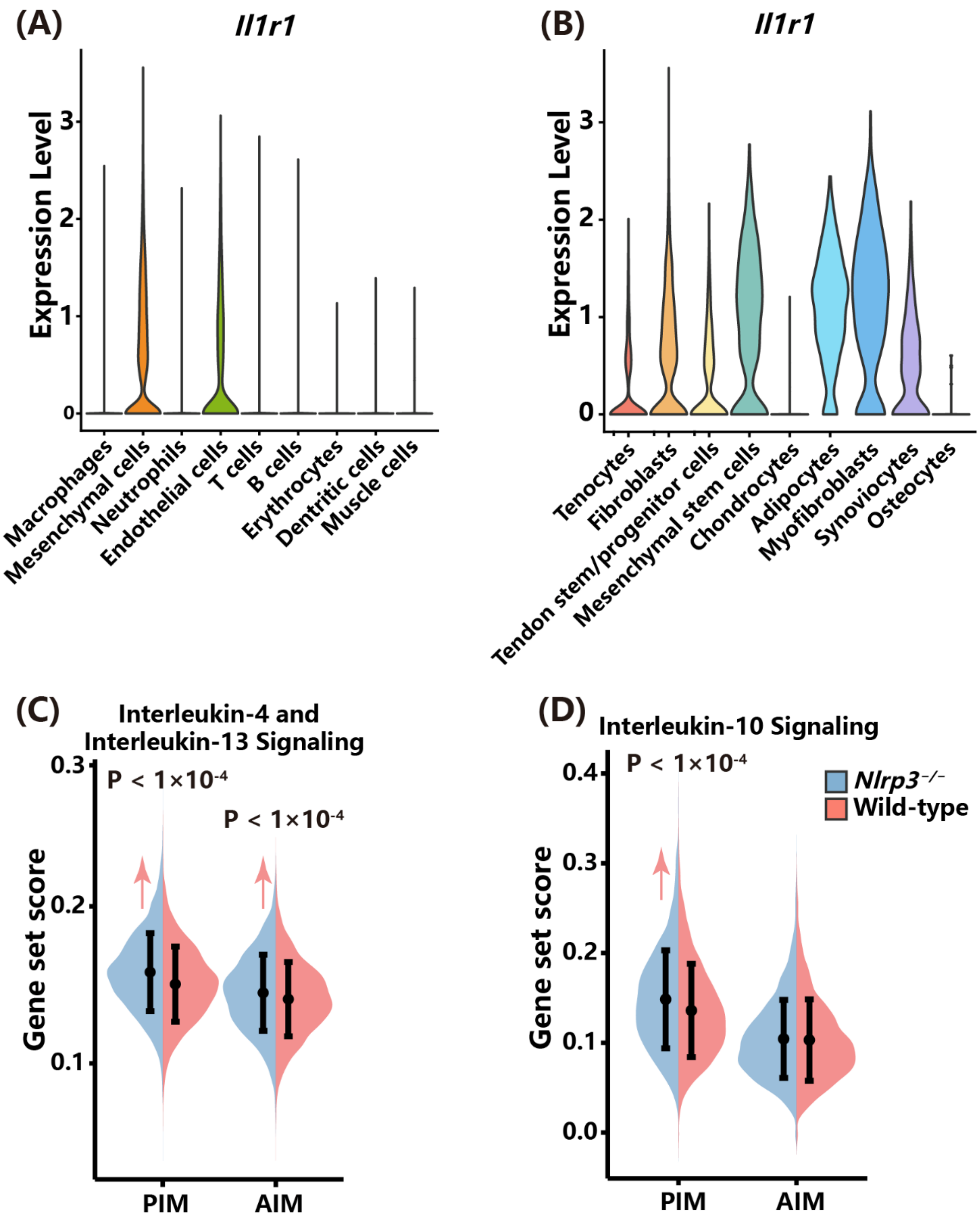

Fig S10. The expression of *Il1r1* and gene set scores in different clusters and subsets. (A) Violin plot of *Il1r1* expression in various clusters. (B) Violin plot of Il1r1 expression in mesenchymal cells subsets. (C and D) Gene set scores of IL-4 and IL-13 signaling, and IL-10 signaling in PIM and AIM in wild-type controls and *Nlrp3*$^{-/-}$ mice. Statistical significance was determined using Student's t-test.

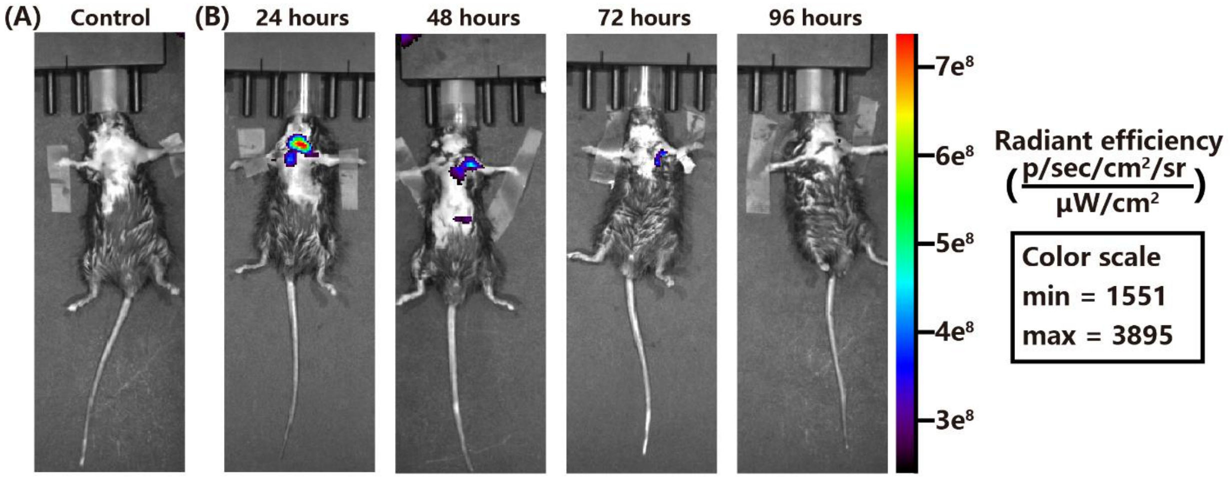

Fig S11. *In vivo* imaging of IL-1β neutralizing antibodies. (A) Mouse without injection was used as control. (B) Neutralizing antibodies were injected into the joint cavity at 3 dpi and visualized at 24, 48, 72, and 96 hours after injection.

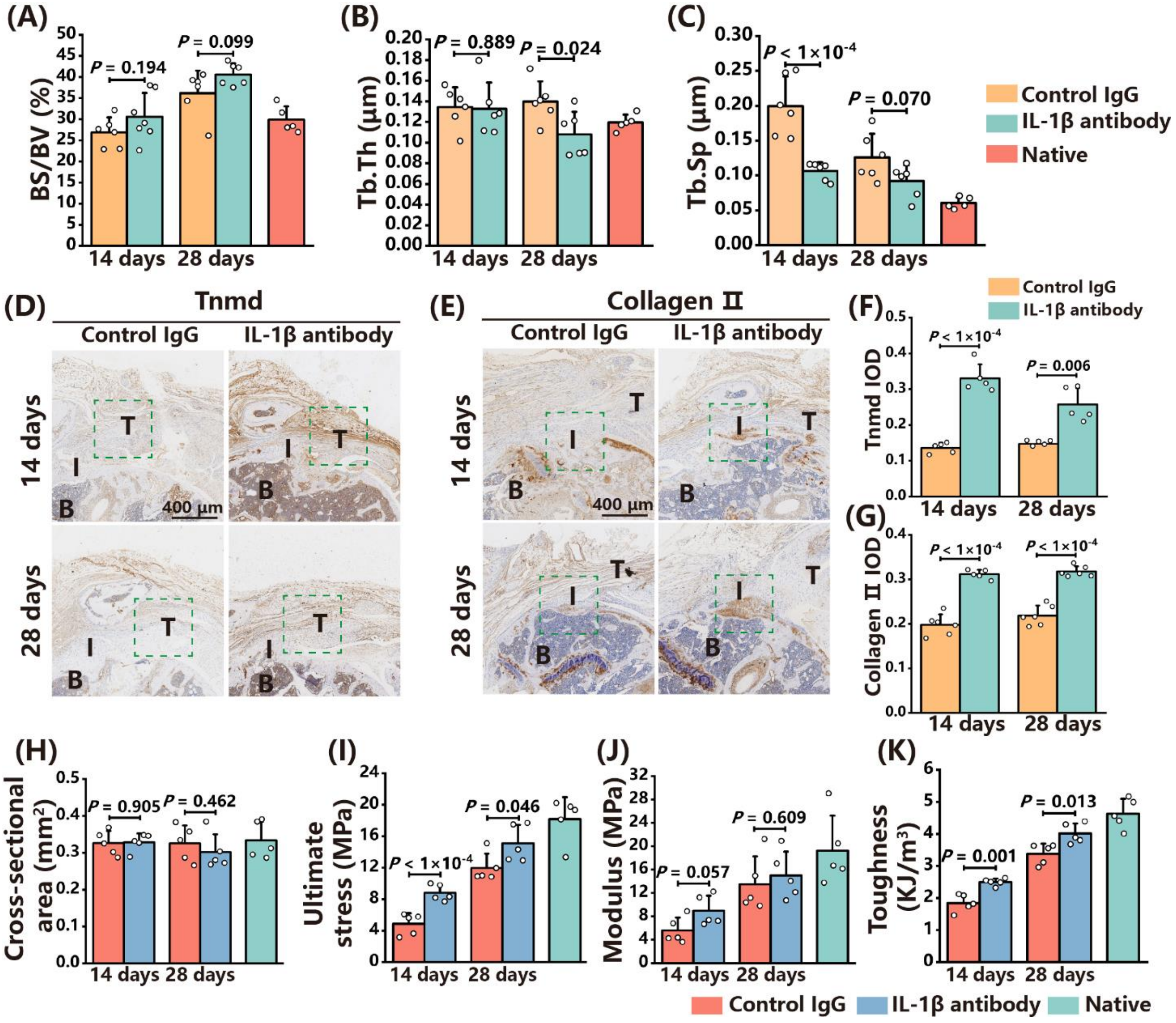

Fig S12. Neutralizing IL-1β accelerates enthesis regeneration. (A-C) Quantitative analysis of BS/BV, Tb.Th, and Tb.Sp of the enthesis. (D-G) Immunohistochemical staining and IOD of Tnmd and collagen II in injured enthesis at 14 and 28 dpi. Green dashed squares represent areas used for quantification. (H-K) Cross-sectional area, ultimate stress, modulus, and toughness of the enthesis in mice with IL-1β neutralizing antibodies or control IgG injection at 14 and 28 dpi. T: tendon, I: tendon-to-bone interface, B: bone. Data are presented as means ± SD. Statistical significance was determined using one-way analysis of variance (ANOVA) with Tukey’s multiple comparisons test and Student’s t-test.

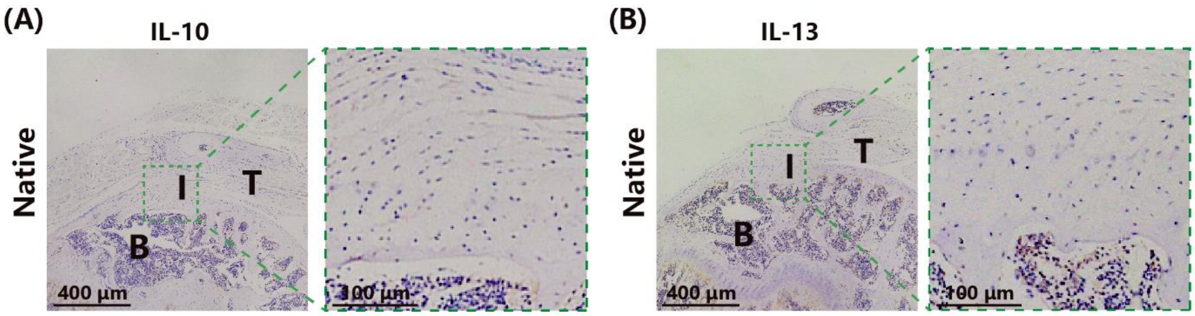

Fig S13. IL-10 and IL-13 expression in native enthesis. (A and B) Expression of IL-10 and IL-13 in native enthesis. Green dashed squares represent the enlarged images of the enthesis. T: tendon, I: tendon-to-bone interface, B: bone.

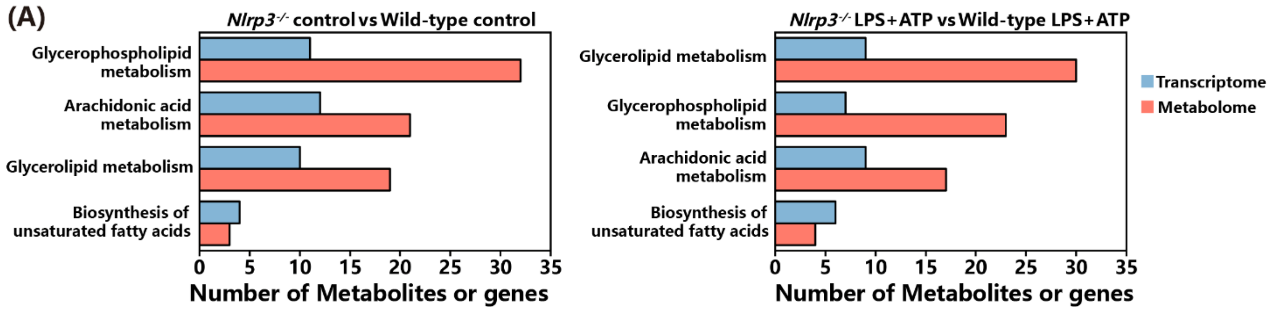

Fig S14. Differential metabolites and genes in biological processes. (A) The number of differential metabolites and genes in GO terms.

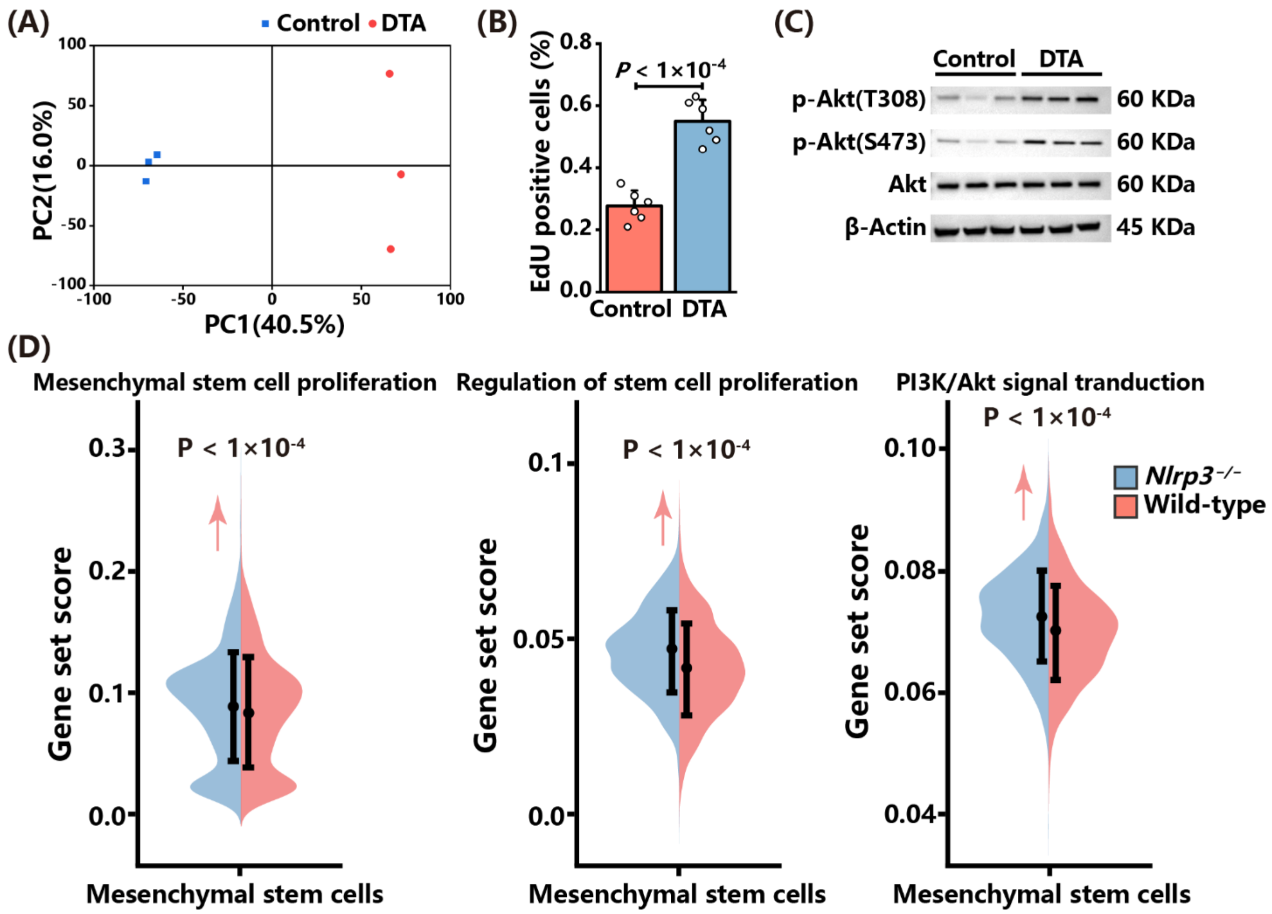

Fig S15. Docosatrienoic acid boosts cell proliferation and enthesis regeneration. (A) PCA of RNA-sequencing of BMSCs with or without DTA treatment. (B) EdU positive cell ratio of BMSCs. (C) Western blot of Akt, p-Akt (S473), p-Akt (T308), and β-actin. (D) Gene set scores of mesenchymal stem cell proliferation, regulation of mesenchymal stem cell proliferation, and PI3K/Akt signal transduction in mesenchymal stem cells in wild-type controls and *Nlrp3*$^{-/-}$ mice. Statistical significance was determined using Student's t-test.

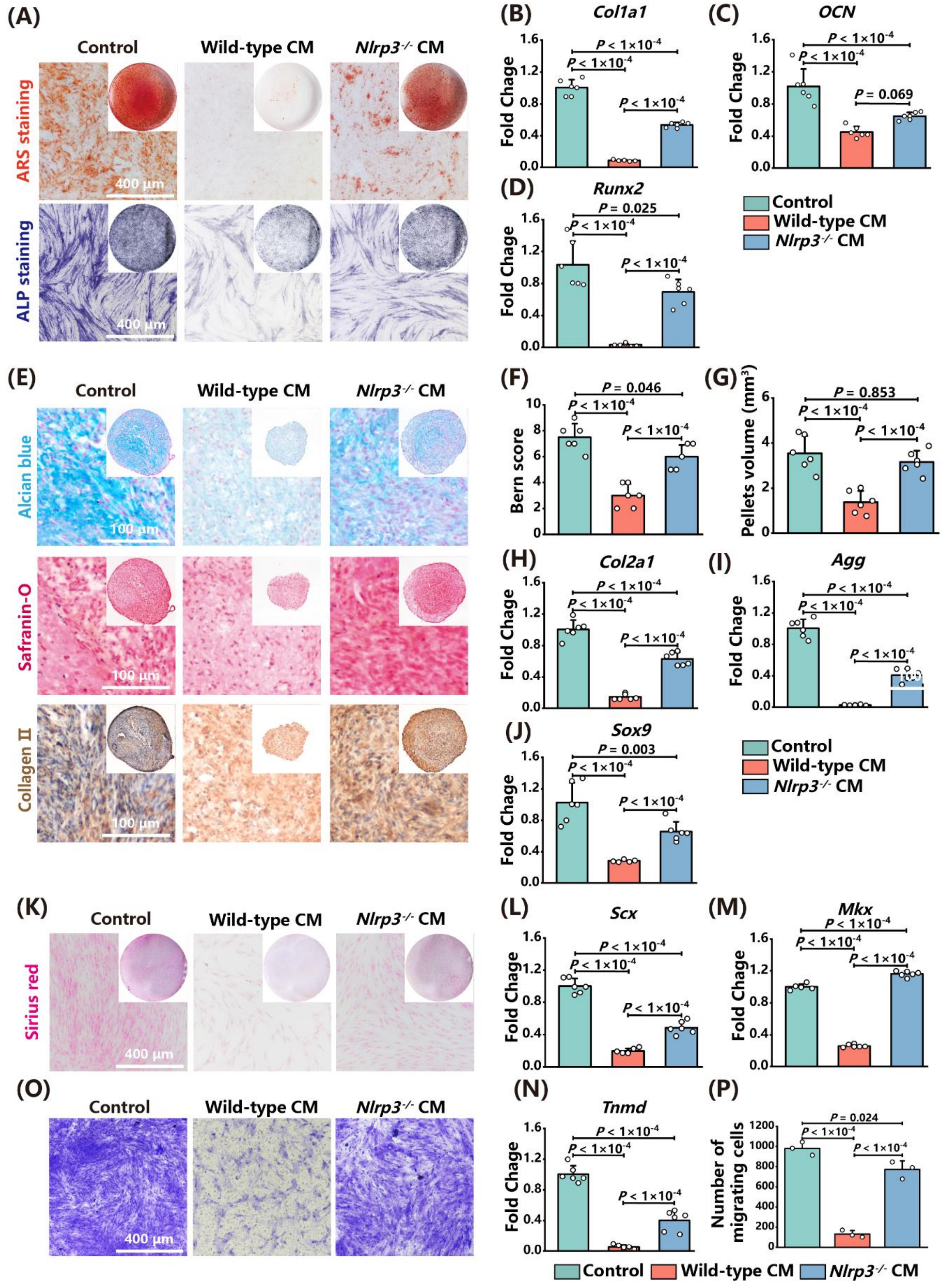

Fig S16. NLRP3 inflammasomes-mediated secretome suppresses the differentiation and migration of stem cells. (A) Alizarin Red and alkaline phosphatase staining of BMSCs cultured with control osteogenic medium, osteogenic medium containing wild-type and *Nlrp3*$^{-/-}$ CM. (B to

D) Relative mRNA expression levels of osteogenic marker genes *Col1a1*, *OCN*, and *Runx2* in BMSCs cultured with corresponding osteogenic medium. (E) Alcian blue, Safranin-O, and collagen II immunohistochemical staining of BMSCs pellets cultured with control chondrogenic medium, and chondrogenic medium containing wild-type or *Nlrp3*$^{-/-}$ CM. (F and G) Bern score and volume of BMSCs pellets cultured with corresponding chondrogenic medium. (H to J) Relative mRNA expression levels of chondrogenic marker genes *Col2a1*, *Aggrecan*, and *Sox9* in BMSCs cultured with corresponding chondrogenic medium. (K) Sirius red staining of TSPCs cultured with control tenogenic medium, and tenogenic medium containing wild-type or *Nlrp3*$^{-/-}$ CM. (L to N) Relative mRNA expression levels of tenogenic marker genes *Tnmd*, *Scx*, and *Mkx* in TSPCs cultured with corresponding tenogenic medium. (O and P) Transwell assay and quantification of TSPCs in corresponding tenogenic medium. Data are presented as means ± SD. Statistical significance was determined using one-way analysis of variance (ANOVA) with Tukey's multiple comparisons test and Student's t-test.

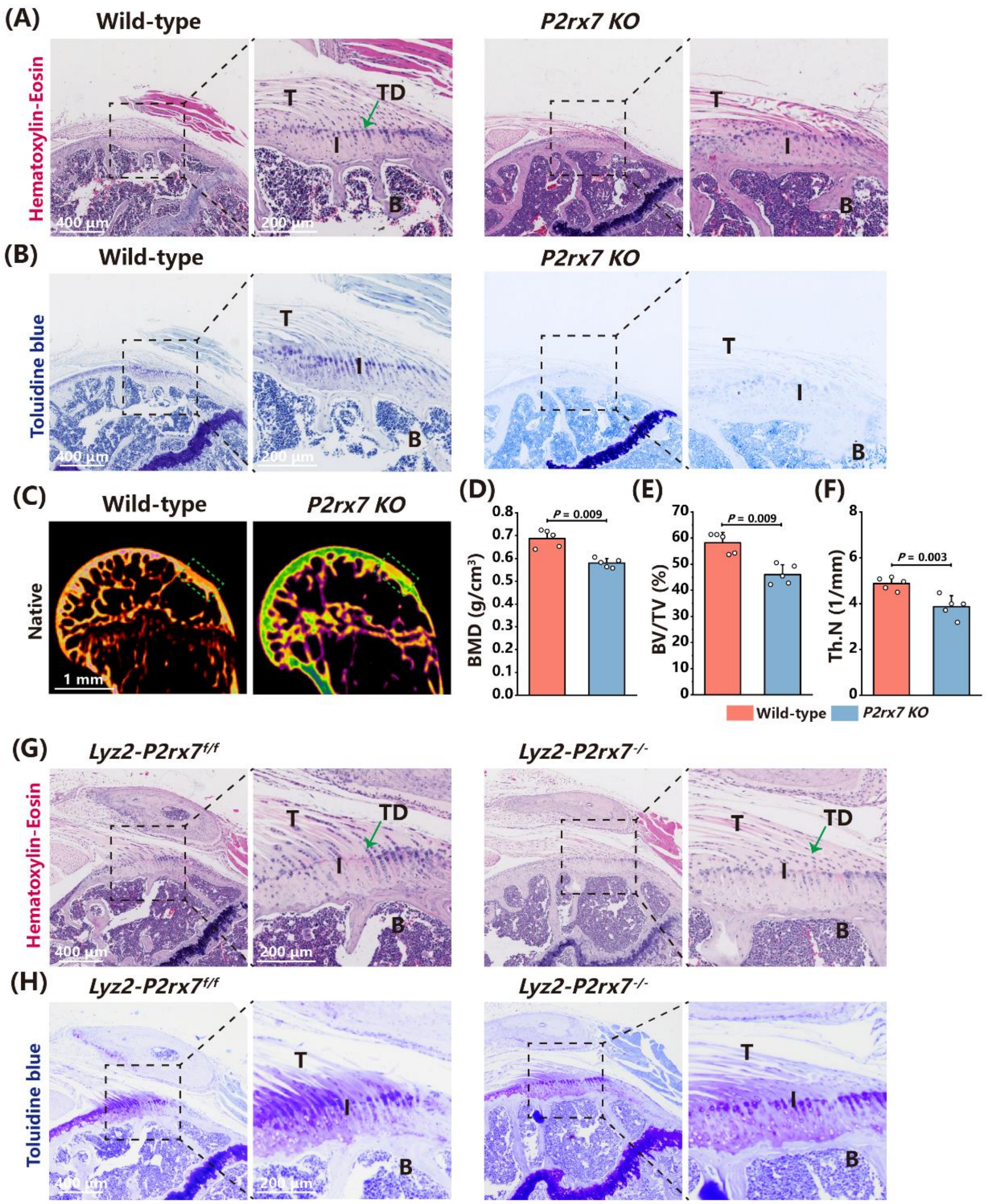

Fig S17. P2X7R participates in the development of the enthesis. (A and B) H&E and toluidine blue staining of the enthesis in wild-type and *P2rx7* knockout mice. (C) Micro-CT of native enthesis in wild-type and *P2rx7* knockout mice. Green dashed squares represent the area of the enthesis. (D to F) Quantitative analysis of BMD, BV/TV, and Tb. N of native enthesis in wild-type and *P2rx7* knockout mice. (G and H) H&E and Toluidine blue staining of the enthesis in *Lyz2-P2rx7*$^{f/f}$ and *Lyz2-P2rx7*$^{-/-}$ mice. T: tendon, I: tendon-to-bone interface, B: bone, TD: tidemark. Data are presented as means ± SD. Statistical significance was determined Student's t-test.

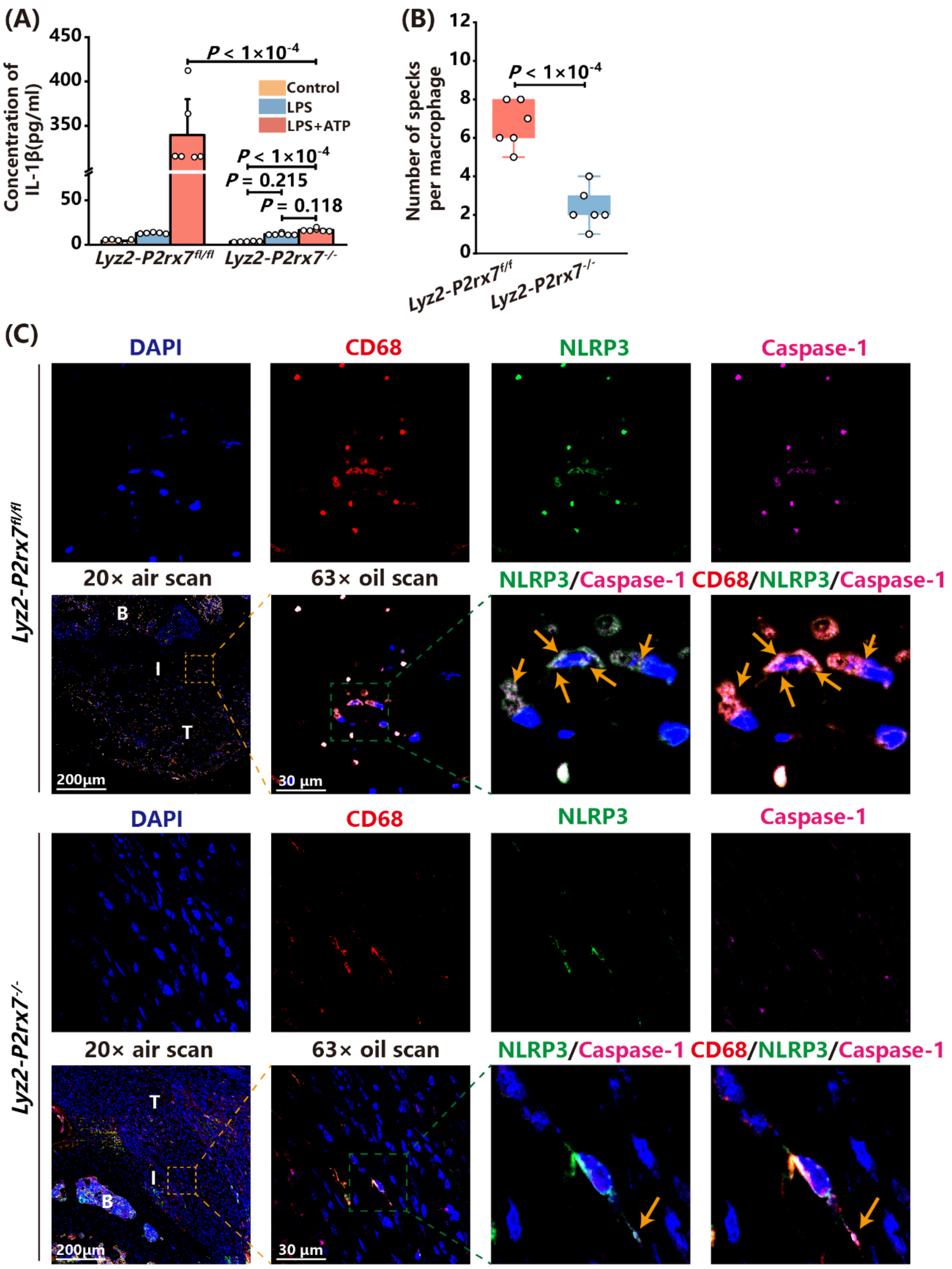

Fig S18. Conditional knockout of *P2rx7* in myeloid cells reduces NLRP3 inflammasomes activation after enthesis injury. (A) IL-1β concentration in the supernatant of BMDMs isolated from *Lyz2-P2rx7*$^{f/f}$ and *Lyz2-P2rx7*$^{-/-}$ mice. (B) Number of specks per macrophage in the injured enthesis. (C) Immunofluorescence staining of CD68 (red), NLRP3 (green), and Caspase-1 (magenta)

in the injured enthesis. Orange and green dashed squares represent the enlarged images of the enthesis. Arrows indicate the colocalization of NLRP3 and Caspase-1. T: tendon, I: tendon-to-bone interface, B: bone. Data are presented as means ± SD. Statistical significance was determined using one-way analysis of variance (ANOVA) with Tukey's multiple comparisons test and Student's t-test.

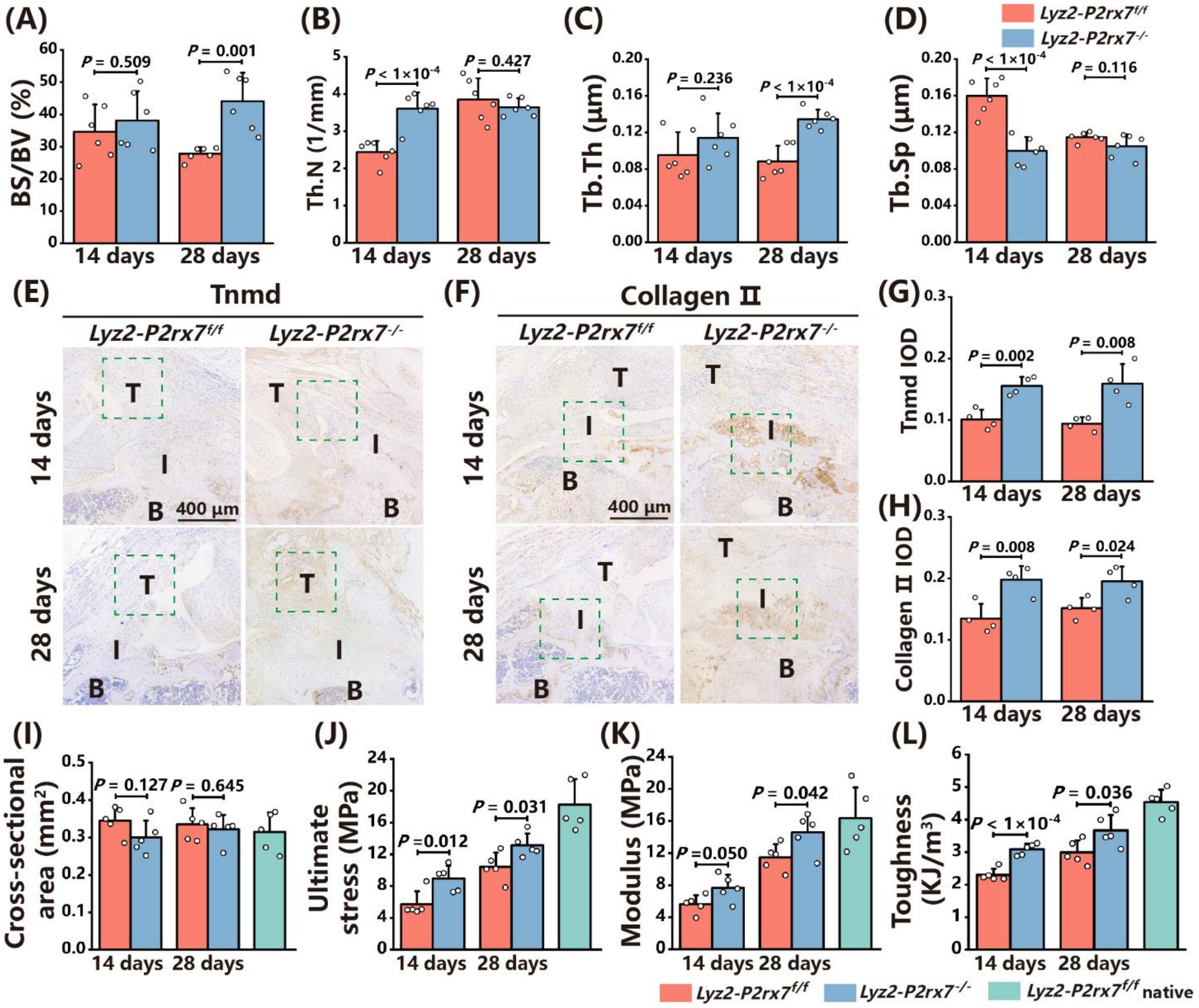

Fig S19. Conditional knockout of *P2rx7* in myeloid cells accelerates enthesis regeneration. (A-D) Quantitative analysis of BS/BV, Tb.N, Tb.Th, and Tb.Sp of the enthesis. (E-G) Immunohistochemical staining and IOD of Tnmd and collagen II in injured enthesis at 14 and 28 dpi. Green dashed squares represent areas used for quantification. (H-K) Cross-sectional area, ultimate stress, modulus, and toughness of the enthesis in *Lyz2-P2rx7*$^{f/f}$ and *Lyz2-P2rx7*$^{-/-}$ mice at 14 and 28 dpi. T: tendon, I: tendon-to-bone interface, B: bone. Data are presented as means ± SD. Statistical significance was determined using one-way analysis of variance (ANOVA) with Tukey's multiple comparisons test and Student's t-test.

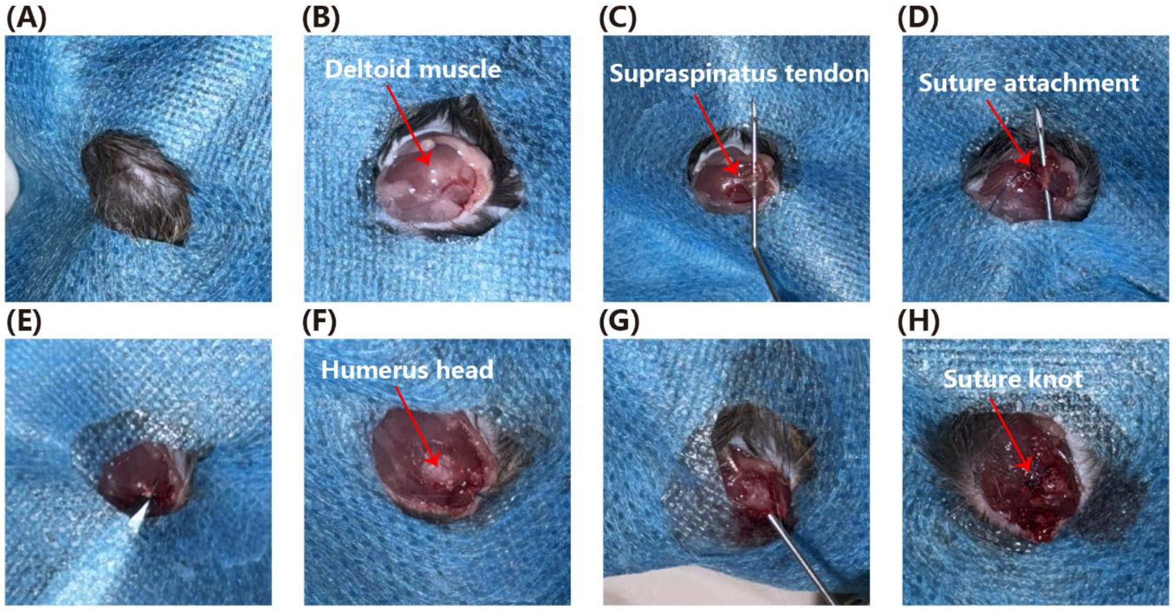

Fig S20. Surgical procedures for the mouse model. (A and B) A 2 cm incision was made to expose the deltoid muscle. (C) The proximal deltoid was peeled off to expose the supraspinatus tendon, and the supraspinatus tendon was elevated with a tissue probe. (D) Suture attachment was generated with a 6-0 Prolene suture with 9.0 taper-point needles in a modified Kessler suture pattern. (E) The supraspinatus tendon was then transected with a #11 blade close to the great tuberosity. (F) The remnant enthesis was debrided with a scalpel. (G) Subsequently, two crossing bone tunnels were created using a 27-gauge needle. (H) The 6-0 Prolene suture was then passed through each tunnel and tied in a knot to repair the rotator cuff.

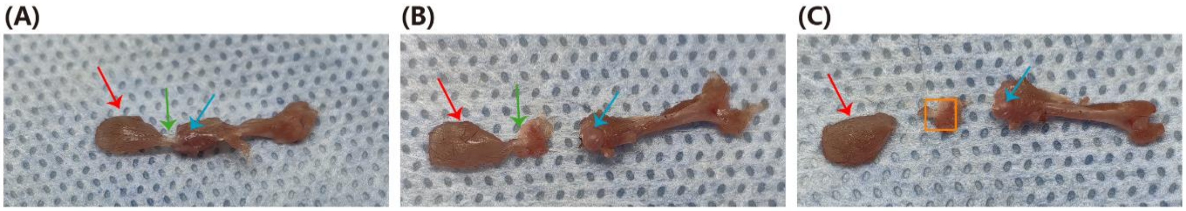

Fig S21. Separation of enthesis tissue. (A) The supraspinatus-humeral head complex was obtained. (B) The cortical bone of the greater tuberosity of the humeral head was separated. (C) Enthesis tissue was isolated. Red, green, and blue arrows represent supraspinatus muscle, supraspinatus tendon, and humeral head, respectively. The orange square represents enthesis tissue.

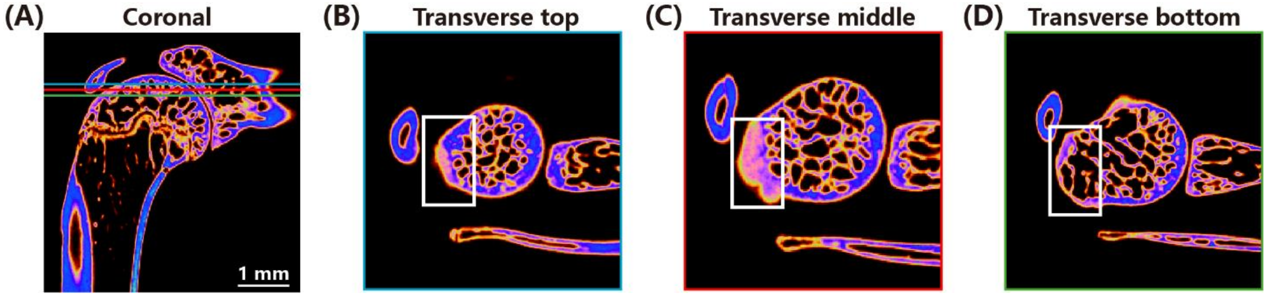

Fig S22. The definition of the enthesis ROI. (A) The coronal image of the humeral head has three lines representing the top, middle, and bottom transverse images, respectively. (B to D) The top, middle, and bottom transverse images of the enthesis. The white rectangle represents the selected area in each image.

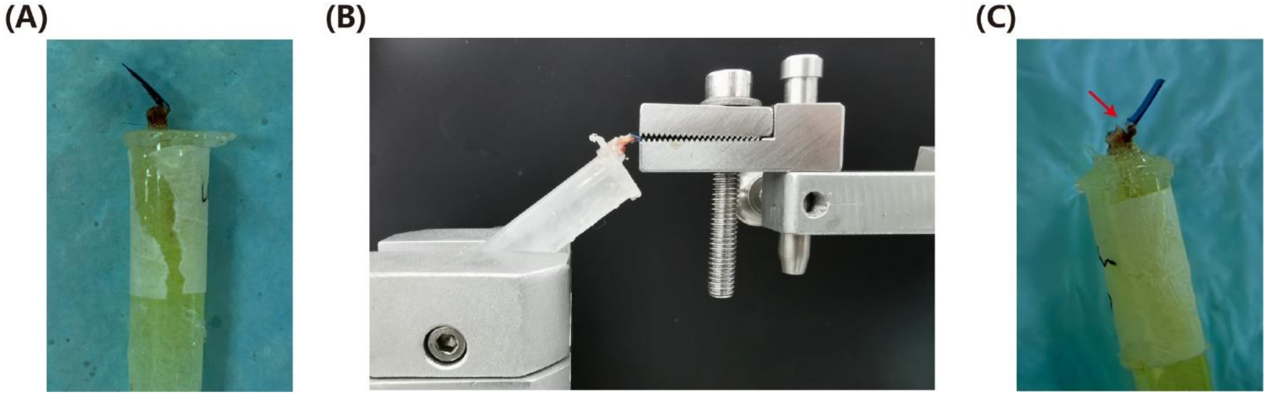

Fig S23. Biomechanical tests. (A) Sample preparation before testing. (B) Biomechanical testing scenarios. (C) The rupture of the enthesis. The red arrow represents the breaking point.

Table S1. Primer sequence used for genotyping of *Nlrp3*$^{-/-}$, *P2rx7*$^{-/-}$, *Lyz2-Cre* and *P2rx7* $^{fl/fl}$ mice

	Sequence (5’→3‘)	Primer type
Nlrp3$^{-/-}$	TGC CTG CTC TTT ACT GAA GG	Mutant
	TCA GTT TCC TTG GCT ACC AGA	Wild type
P2rx7 KO	GCC AGA GGC CAC TTG TGT AG	Mutant
	TCA CCA CCT CCA AGC TCT TC	Wild type
Lyz2-Cre	ACC GGT AAT GCA GGC AAA T	Mutant
	GTC ACT CAC TGC TCC CCT GT	Wild type
P2rx7$^{fl/fl}$	GATTAAAGGCATGTGCCACCACA	Mutant
	CATCTCACCTATGGAGGTGAAATTCA	Wild type

Table S2. Modified tendon maturing score

	1	2	3	4
Cellularity	Marked	Moderate	Mild	Minimal
Vascularity	Marked	Moderate	Mild	Minimal
Continuity	-25%	25-50%	50%-75%	75%-
Fibrocartilage	-25%	25-50%	50%-75%	75%-
Tidemark	-25%	25-50%	50%-75%	75%-

Percentage was relative values compared to native enthesis.

Table S3. Antibodies used in this research

Reactivity	Supplier	Product number	Application
CD68	Abcam	ab955	IHC IF
iNOS	Abcam	ab283655	IF
IL-1β	Abcam	ab283818	IHC
NLRP3	Proteintech	68102-1-lg	IHC IF
Caspase-1	ABclonal	A21296	IHC IF
Tnmd	Abcam	ab203676	IHC IF
CollagenII	Arigo	ARG20787	IHC IF
IL-10	ABclonal	A22083	IHC
IL-13	ABclonal	A2089	IHC
CD31	Abcam	ab182981	IF
α-SMA	Proteintech	14395-1-AP	IF
Pdgfrα	Abcam	Ab203491	IF
P2X7R	Proteintech	28207-1-AP	IHC IF
β-actin	CST	4967S	WB
Akt	CST	2920S	WB
p-Akt（S473)	CST	2965S	WB
p-Akt（T308)	CST	4060S	WB

Table S4. Bern score of pellets

Scoring categories	Score
A. Uniformity and darkness of Safranin O-fast green stain	
No stain	0
Weak staining of poorly formed matrix	1
Moderately even staining	2
Eevn darl stain	3
B. Distance between cells/amount of matrix accumulated	
High cell densities with no matrix in between	0
High cell densities with little matrix in between	1
Moderate cell density with matrix	2
Low cell density with moderate distance between cells and an extensive matrix	3
C. Cell morphologies represented	
Condensed/necrotic/pycnotic bodies	0
Spindle/fibrous	1
Mixed spindle/fibrous with rounded chondrogenic morphology	2
Majority rounded/chondrogenic	3

Table S5. Primer sequence used for RT-qPCR

Gene	Primer sequence	
Col1a1	Forward	GAGGGCCAAGACGAAGACATC
	Reverse	CAGATCACGTCATCGCACAAC
OCN	Forward	CACTCCTCGCCCTATTGGC
	Reverse	CCCTCCTGCTTGGACACAAAG
Runx2	Forward	TGGTTACTGTCATGGCGGGTA
	Reverse	TCTCAGATCGTTGAACCTTGCTA
Agg	Forward	ACTCTGGGTTTTCGTGACTCT
	Reverse	ACACTCAGCGAGTTGTCATGG
Col2a1	Forward	TGGACGATCAGGCGAAACC
	Reverse	GCTGCGGATGCTCTCAATCT
Sox9	Forward	AGCGAACGCACATCAAGAC
	Reverse	CTGTAGGCGATCTGTTGGGG
Scx	Forward	CTGGCCTCCAGCTACATTTCT
	Reverse	GTCACGGTCTTTGCTCAACTT
Tnmd	Forward	ACACTTCTGGCCCGAGGTAT
	Reverse	GACTTCCAATGTTTCATCAGTGC
Mkx	Forward	GGGGAGCCGTGCTTTTTGA
	Reverse	GCCTTACCTTCCCTCCATTCTG
Il1b	Forward	GCAACTGTTCCTGAACTCAACT
	Reverse	ATCTTTTGGGGTCCGTCAACT
Nlrp3	Forward	ATTACCCGCCCGAGAAAGG
	Reverse	TCGCAGCAAAGATCCACACAG

Caspase-1	Forward	ACAAGGCACGGGACCTATG
	Reverse	TCCCAGTCAGTCCTGGAAATG
P2rx7	Forward	GACAAACAAAGTCACCCGGAT
	Reverse	CGCTCACCAAAGCAAAGCTAAT
GAPDH	Forward	AGGTCGGTGTGAACGGATTTG
	Reverse	TGTAGACCATGTAGTTGAGGTCA
